# Microbiome Dependent Regulation of T_regs_ and Th17 Cells in Mucosa

**DOI:** 10.3389/fimmu.2019.00426

**Published:** 2019-03-08

**Authors:** Pushpa Pandiyan, Natarajan Bhaskaran, Mangge Zou, Elizabeth Schneider, Sangeetha Jayaraman, Jochen Huehn

**Affiliations:** ^1^Department of Biological Sciences, School of Dental Medicine, Case Western Reserve University, Cleveland, OH, United States; ^2^Experimental Immunology, Helmholtz Centre for Infection Research, Hamburg, Germany; ^3^Cluster of Excellence RESIST (EXC 2155), Hannover Medical School, Hannover, Germany

**Keywords:** microbiome, mucosa, T_reg_, mucosal immunity, inflammation, Th17, antibiotics, resident microbes

## Abstract

Mammals co-exist with resident microbial ecosystem that is composed of an incredible number and diversity of bacteria, viruses and fungi. Owing to direct contact between resident microbes and mucosal surfaces, both parties are in continuous and complex interactions resulting in important functional consequences. These interactions govern immune homeostasis, host response to infection, vaccination and cancer, as well as predisposition to metabolic, inflammatory and neurological disorders. Here, we discuss recent studies on direct and indirect effects of resident microbiota on regulatory T cells (T_regs_) and Th17 cells at the cellular and molecular level. We review mechanisms by which commensal microbes influence mucosa in the context of bioactive molecules derived from resident bacteria, immune senescence, chronic inflammation and cancer. Lastly, we discuss potential therapeutic applications of microbiota alterations and microbial derivatives, for improving resilience of mucosal immunity and combating immunopathology.

## Introduction

Mammals harbor a highly diverse microbiome of at least 1000 species, and an astounding number of 10–100 trillion microbial cells, co-existing in a remarkable balance with the host immune system. Healthy human microbiome is mostly bacteria although other microbial domains such as archaea, viruses, and eukaryotes (principally fungi and protists) are also present (1). While these microbes are distributed in skin, and mucosa of ocular, nasal, oral, eye, and reproductive organs, gastrointestinal (GI) tract mucosa is the major reservoir of resident microbes in terms of abundance and species diversity (2, 3). The human colon harbors approximately 3.8 × 10^13^ microorganisms, followed by skin in the range of ~10^11^(4). Since the resurgence of microbiome research in recent years, there has been a sharp increase in understanding of how resident microbiome shapes immunity, health and disease of humans. Only a perennial holiday on a lonely island could excuse an immunologist's incognizance on intimate interrelationships between intestinal microbiota and immune balance. Direct crosstalk between resident microbes and host immune cells in mucosa emerges as a pivotal determinant of such an immune balance. Dysbiosis of resident microbes has strong association with a number of immunological disorders, including opportunistic and pathogenic infections (5–13).

Mucosal immune system has not only evolved to protect the mucosal barrier surface against external insults, it has also co-evolved with resident microbes in an interdependent harmonious relationship with them (14–21). The resulting immune balance is crucial to drive optimal immune responses without causing an over-exuberant inflammation (22–25). Past few decades have seen that an increase in hyper-hygiene mentality, mindless use of antibiotics and diet changes, have led to reduced diversity and impaired resilience in resident microbiota (26). Consequently, a disruption in aforementioned immune balance leads to rise in autoimmune and inflammatory disorders. Therefore, understanding the mechanisms of these mutualistic relationships between resident microbiota and different components of innate and adaptive immunity is vital to our understanding of immune diseases. Although gut microbiota in laboratory mice and humans differ significantly, murine models have provided a powerful tool to explore host-microbiota-pathogen interactions in mucosa (27, 28). Here we review the effects of resident microbiota on T_regs_ and Th17 cells, important players in determining immune balance, mucosal barrier integrity and host protective functions in mucosa. These cells mucosa can develop in mucosa independent of commensal microbiota. For example, there is evidence in germ free mice that T_reg_ cells can be induced by dietary antigens from solid food (29). These T_reg_ cells are of limited life span, but are distinguishable from microbiota- induced T_reg_ cells and capable of repressing inadvertent immune responses to ingested protein antigens. Similarly, in oral mucosa, mechanical damage from mastication of food induces barrier protective Th17 cells, independent of oral commensal microbiota under homeostatic conditions (30). However, dysbiosis can lead excessive Th17 cells and lead to periodontal inflammation (31). Thus, while it is known that these cells can develop independent of microbiota, resident bacterial dysbiosis is strongly associated with alterations in these cells, causing mucosal inflammation seen in many diseases including HIV immunopathogenesis (32–41). Although other cells also play important roles in mucosal tolerance and immunity, we will not review them here.

## T_REGS_ and Th17 Cells in Mucosa Under Steady State-Conditions

Majority of the studies on mucosa-microbiota interactions discuss GI tract. Indeed, GI mucosa harbors by far the largest and most diverse microbiota, as well as abundant and dynamic population of T_regs_ and Th17 cells. T_regs_ are defined by the expression of CD25 and Foxp3, and are predominantly known for their immunosuppressive properties. These cells also express other molecules such as Cytotoxic T Lymphocyte Antigen-4 (CTLA-4), PD-1, interleukin 10 (IL-10), transforming growth factor beta 1(TGF-β1), and amphiregulin. Each of the aforementioned proteins has been shown to be either important, or dispensable for different mechanisms of T_reg_-mediated immunosuppression. Divergent conclusions derived from various T_reg_ mechanism investigations have been strikingly similar to those in the popular parable of the “Blind men and an elephant.” It is now increasingly clear that suppressive and non-suppressive functions of Foxp3^+^ cells are largely variable, depending on local tissues, disease phenotypes, responding effector cells, and cytokine milieu (42–49).

While CD4^+^ effector T cell responses contribute to overt intestinal inflammation, T_regs_ are associated with controlling immunopathology (42, 43, 50). It is well known that T_regs_ are also pivotal for commensal tolerance (51–53). There have been contentions regarding the T_regs_ found in colon mucosa (colon T_regs_; cT_regs_); whether they develop in thymus (thymic T_regs;_ tT_regs_), or periphery (peripheral T_regs_; pT_regs_). The usage of Nrp-1 and Helios as markers of tT_regs_, and the extent to which the TCR repertoire of cT_reg_ overlaps with that of tT_regs_ have been debated (54, 55). Nevertheless, it is well established that cT_regs_ require the presence of microbiota for their development, sustenance and function (56–58). There is also evidence that mucosal sites are the primary sites of development and maintenance of pT_regs_ (59–61). First formal proof for the requirement of microbiota for the induction and maintenance of intestinal T_regs_ was provided by studies using germ-free (GF) animal models. GF mice show a several-fold reduction in the frequency of Helios^−^ T_regs_, when compared with conventionally housed specific pathogen free (SPF) mice. Association of individual bacterial isolates or defined consortia in GF mice is sufficient to induce intestinal T_regs_ (56, 57). Even antibiotic treated mice, which show depletion in resident microbiota correlating with a drastic reduction in the frequency of T_regs_, lend further credence to the positive role of microbiota in sustenance of T_regs_ (53, 55, 62). In addition to commensal tolerance, mucosal T_regs_ have been shown to regulate excessive immune responses during infections (43, 63–65). Recently, they are also shown to accumulate in other tissues and provide functions such as non-suppressive tissue repair functions in muscle (66). While T_regs_ play diverse and often opposite roles in mucosal infections (Table 1), effects of microbiome on T_regs_ during these infections are largely ignored in many studies.

**Table 1:** Foxp3^+^T_reg_ functions in mucosal infections.

**Pathogen**	**T_**reg**_ manipulation**	**Outcome**
**BACTERIA**		
*Listeria monocytogenes*	T_regs_ cause increased pathogen burden (67)	Detrimental
*Salmonella enterica*	Foxp3+ cell ablation accelerates bacterial clearance (68)	Detrimental
*Aggregatibacter actinomycetemcomitans*	T_regs_ attenuate experimental periodontitis progression (69)	Protective
*Yersinia Enterocolitica*	T_regs_ reduce pathogenic burden and attenuate inflammation (70)	Protective
**VIRUSES**		
HIV	Early interference with the T_reg_'s suppressive function worsened infection and inflammation (71, 72)	Protective
	T_regs_ are preserved in elite controllers in humans (73)	Protective
	T_regs_ suppress anti-viral CD8 responses (74)	Detrimental
	Foxp3+ cell ablation accelerates mortality and increases viral load (197)	Protective
Herpes simplex virus 2	Foxp3+ cell ablation increases mortality (75)	Protective
West Nile virus		
**PARASITES**		
*Toxoplasma gondii*	Loss of Foxp3+ T_reg_ cells results in fatal pathology (76)	Protective
*Toxoplasma gondii*	Loss of Foxp3+ T_reg_ cells results in pathology (77)	Protective
*Toxoplasma gondii*	Loss of Foxp3+ T_reg_ cells results in pathology (78)	Protective
*Heligmosomoides polygyrus*	No changes in pathogen burden with T_reg_ ablation (79)	No effect
*Leishmania major*	T_regs_ promote increased pathogen burden (80).	Detrimental
*Schistosoma mansoni*	CD4^+^CD25^+^ depletion increases inflammation (81)	Protective
**FUNGUS**		
*Candida albicans*	CD4+CD25+T_regs_ regulate immunopathology in Th1 mediated gastrointestinal/disseminated Candidiasis (82)	Protective
	CD4+CD25+Foxp3+T_regs_ promote Th17 antifungal immunity and dampen immunopathology (41, 83)	Protective
	T_regs_ regulate immunopathology (84)	
	T_regs_ suppress pulmonary hyperinflammation (85)	
*Aspergillus fumigatus*		Protective
*Pneumocystis carinii*		Protective
**MYCOBACTERIA**		
*Mycobacterium tuberculosis*	Selective depletion of T_regs_ reduces pathogen burden (86).	Detrimental
	Foxp3+ cells induce resistance to TB lesions (87)	Protective

Th17 cells are RORγt^+^, CCR6^+^, IL-17A^+^, IL-17F^+^, with some cells expressing IL-21 and IL-22, and have been implicated both in mucosal barrier functions. Th17 cells are an important subset of effector T cells that are protective during extracellular bacterial and fungal invasion (83, 88–91). However, excessive Th17 responses are also associated with a variety of pathogenic conditions, depending on the pro-inflammatory cytokines they co-produce (30, 91–95). Littman and colleagues showed for the first time that commensal microbiota play important roles in the development of intestinal Th17 cells (22, 53, 96–100). Th17 development and differentiation is controlled by cytokine and epigenetic regulation (91, 92, 101, 102), but the mechanistic details of microbiome dependent control of Th17 development during mucosal infection is largely unclear.

## Impact of Microbiome on T_REGS_ and Th17 Cells During GI Infection and Inflammation

“Healthy” GI microbiota is mainly composed of the phyla Actinobacteria, Bacteroidetes, Firmicutes, Fusobacteria, Proteobacteria, and Verrucomicrobia. Small intestine is dominated by Enterobacteriaceae and Lactobacillaceae, whereas colon contains the members of Bacteroidaceae, Lachnospiraceae, Prevotellaceae, Rikenellaceae, and Ruminococcaceae respectively (3). A number of factors including diverse environmental conditions, intake of diet and medication, as well as host genetic factors determine the dynamic composition of gut microbiota in individuals (103–107). Gut microbiota are capable of restraining the mucosal colonization by enteric pathogens, a process defined as colonization resistance (108). Thus, administration of antibiotics, and altering the resident microbiota during a mucosal infection is known to lead to post-antibiotic expansion of the pathogens. Loss of overall diversity, or even deficit in single group of bacteria can alter the susceptibility to gastrointestinal infections. For example, Clostridium difficile (C. difficile) infection, the most common cause of nosocomial diarrhea is often preceded by antibiotic usage. Colonization of C. difficile in healthy mice in fact requires a pre-exposure to a cocktail of antibiotics to alter the microbiota composition (109). However, mono-colonization of GF mice with a murine isolate from the family Lachnospiraceae could limit the colonization of C. difficile, suggesting that individual bacterial species are sufficient to confer colonization resistance to C. difficile (110). Enhanced susceptibility toward other infections after antibiotic-mediated disruption of the intestinal microbiota composition has also been reported for vancomycin-resistant Enterococcus Spp and Salmonella enterica serovar typhimurium (*S. typhimurium*) (108, 111). Mechanistically, mucosal carbohydrates such as fucose and sialic acid liberated by resident microbiota have been shown to control the growth of enteric pathogens. Antibiotics cause spikes in sugars that can worsen *S. typhimurium* and C. difficile infections (112). Microbiota alterations reduce the numbers of germinal centers in IL21-receptor knockout mice, resulting in diminished IgA^+^ B cells and reduced activation-induced cytidine deaminase in Peyer's patches. These events lead to the expansion of T_regs_ and Th17 cells, and higher bacterial burdens, but dampening of Citrobacter rodentium-induced immunopathology (113). Resident microbiota at mucosal interfaces can govern transmission and progress of parasitic protozoan infections such as Toxoplasmosis and Amoebiasis (114). In the case of Toxoplama gondii infection in mice, reduction of microbiota in the gut by prolonged antibiotic treatment leads to impaired Toll like receptor (TLR)-11 and Myeloid differentiation response 88 (MyD88) signaling and subsequent deficit in Th1 immunity, substantiating that gut commensals serve as natural molecular adjuvants during T. gondii infection (115). In a mouse model of Giardia duodenalis infection, antibiotic induced alteration of the microbiome prevents CD8 T cell activation by G. duodenalis. Conversely, GI infection can also modulate microbiota specific adaptive immunity (116). For example, a pathogenic GI infection, in parallel to specific immune reactions against the pathogen, induces immune responses to commensals and generates long-lived commensal-specific T cells. Thus an adaptive response against commensals is an integral component of mucosal immunity. However, such a commensal specific-adaptive response in a dysbiosis setting can also contribute to excessive inadvertent inflammation. In the context of HIV-1 infection, damages in GI tract and gut microbial translocation (Proteobacterial species) are associated with reduction of systemic and gut/rectal mucosal Th17 cells and T_regs_ (despite increased T_reg_/Th17 ratio) (36, 71, 72, 117, 118). A large body of evidence suggests that increased T_regs_ in circulation correlate to reduced immune activation in HIV+ patients, underlining the anti-inflammatory protective roles of T_regs_ in patients (71–73, 118–125). While combined anti-retroviral (cART) therapy in HIV^+^ patients generally ensures immune reconstitution in the peripheral blood, dysbiosis and T_reg_/Th17 abnormalities persist in gut and other mucosae (41, 126–132). This can present residual inflammation and heightened morbidities in cART treated HIV^+^ patients. However, in cART-treated HIV^+^ patients with elevated levels of immune activation, it is not clear whether altered levels and function of mucosal T_regs_/Th17 cells are associated with local microbial dysbiosis (131), and if these alterations contribute to residual inflammation in HIV disease. Collectively, these findings highlight the role of microbiota in restraining pathogens and inflammation by having significant impact on T_regs_ and Th17 cells.

Alterations in resident microbiota and host immune cells, caused by host genetic makeup also play a role in the pathogenesis of inflammatory bowel diseases (IBD). One of the adaptive arms of immunity that is impacted by such changes is T_regs_ (133). *Bacteroides fragilis* for example, has been found to invade mucosa and cause excessive activation of the host intestinal immune response in genetically susceptible patients (134), while under steady-state conditions the same bacterium can enhance T_reg_ differentiation and ensure intestinal homeostasis. Loss of autophagy protein ATG16L1 in T_regs_ results in aberrant type 2 responses and spontaneous intestinal inflammation (135). It is unclear whether microbiota directly induce the expression of ATG16L1 in T_regs_, but it is evident that ATG16L1 and autophagic process directly promote T_reg_ survival and metabolic adaptation in the intestine. Similarly, other genetic risk variants associated with IBD such as: *NOD2, CARD9, ATG16L1, IRGM and FUT2* significantly influence the gut microbiota changes (136). For example, a decrease in *Roseburia* spp (known acetate to butyrate converters), *Clostridiaceae* family, the genera *Bifidobacterium, Ruminococcus* and *Faecalibacterium* has been observed in patients with IBD. Although many of these communities are strongly implicated in T_reg_ maintenance, direct mechanisms of T_reg_ regulation in the context of these genetic variants and IBD are unclear. Combined deficiency of MyD88 and JH gene, which disrupts innate interactions of immune cells with intestinal microbiota and IgA responses respectively, causes overt inflammation, highlighting the requirement of T_reg_-IgA mediated mechanism in tolerance (51, 137). It has also been shown that microbiota-specific Foxp3^+^ T_reg_ cells can convert to interferon-γ-producing Foxp3^+^ T cells that have a potential to establish mucosal tolerance (138). Disruption of TLR/MyD88 signaling in Foxp3-deficient mice protect them from excessive inflammation at the environmental interfaces of skin, lungs, and intestine, showing that T_regs_ normally also restrain commensal dependent tonic MyD88-dependent pro-inflammatory signals (139). Mice lacking *CLEC7A* gene (Dectin-1), thus having dys-regulated interactions with fungal microbiome (mycobiome) show an increased susceptibility to dextran sulfate sodium (DSS) induced colitis (140). The role of Th17 cells and T_regs_ in this model is unknown. Certain proportion of intestinal T_regs_ co-expresses RORγt, the master transcription factor of the Th17 lineage, with up to 35 % in small intestine and 65 % in colon (141–143). Some of these RORγt^+^ T_reg_ co-produce IL-17A (T_reg_17), and are substantially diminished in GF or antibiotics-treated mice. Mono-association of GF mice with a panel of 22 bacterial species from the human gastrointestinal tract shows that a number of microbes, not only *Clostridiales*, are capable of induce colonic RORγt^+^ T_regs_ (142). Segmented filamentous bacteria (SFB) were only mediocre inducers of RORγt^+^ T_regs_ in that study (142). These studies demonstrate that intestinal RORγt^+^ T_regs_ are highly microbiota-dependent and have functions in promoting host immunity (62). Yet, RORγt is not a perfect marker for pT_regs_, because recent reports show the existence of RORγt^+^ tT_regs_, particularly developing under inflammatory conditions (143–145).

While most studies have focused on in-depth characterization of mechanisms by which microbiota engage to counter-regulate their immunostimulatory properties, the reciprocal effect of T_regs_ on the composition and function of the intestinal microbiota was largely ignored (53, 56, 99, 146, 147). Very recently, analysis of mice harboring a reduced number of TGF-β-dependent pT_regs_ demonstrated numerous underrepresented metabolic processes and a limited overall diversity of the microbiome, including a significant reduction of *Lactobacillus johnsonii* and *Mucispirillum schaedleri* (148). Mechanistically, it was confirmed that the impaired pT_reg_ generation could adversely affect the microbiota niche by elevating type 2 immune responses in the host, thereby declining the microbiota abundance during the process of community assembly. In conclusion, the presence of pT_regs_ in the intestinal immune system has a strong impact on the composition and function of the intestinal microbiota. Similarly, IL-17F deficiency induces T_reg_ cells in the colon and modifies the composition of the intestinal microbiota and mediates protection against colitis (149). Taken together, two-way interactions between resident microbiota and host intestinal immunity confer intestinal tolerance and immunomodulation.

## Impact Of Microbiota on T_REGS_ and Th17 Cells in Oral Mucosa

**Figure 1:**
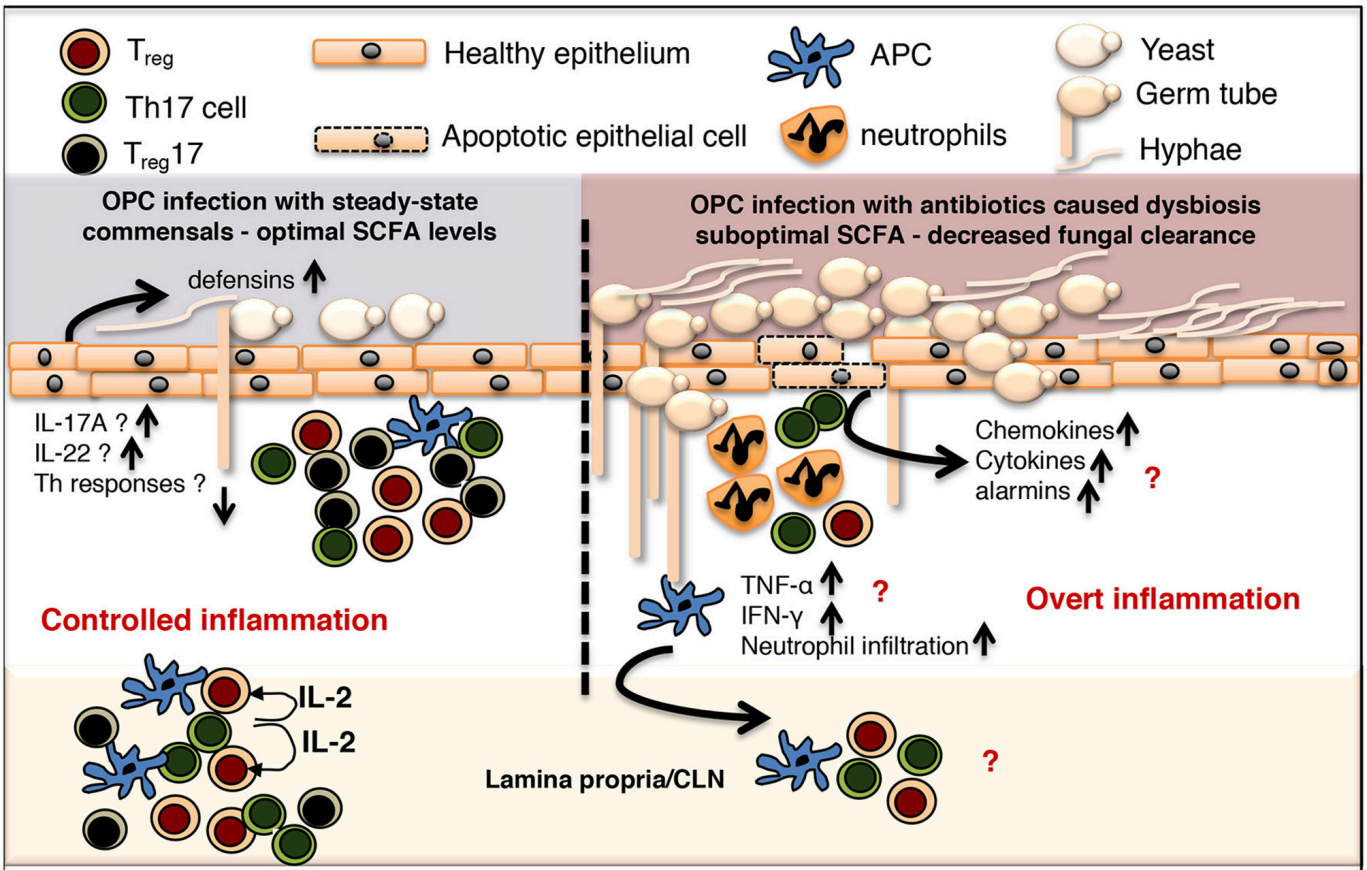
Controlled commensal bacteria/T_reg_/T_reg_17/Th17 cell interplay functions as a switch between protective immunity and overt inflammation in oral mucosa. OPC, Oropharyngeal candidiasis; SCFA, short chain fatty acid; CLN, cervical lymph node; APC, antigen presenting cells.

Oral microbiome is vital to maintaining both oral and systemic immune homeostasis because oral mucosa is the primary gateway for the GI tract, the biggest component of the immune system (150). While a vast majority of microbiota studies has focused on intestinal mucosae and their interactions with gut microbiota, little is known about oral mucosal microenvironment colonized with a large array of resident microbes, which is structurally and functionally distinct from the GI tract (151–160). *Actinobacteria, Bacteroidetes, Firmicutes, Fusobacteria*, and *Proteobacteria* are the major phyla accounting for ~96–99% of the oral microbiome, while SR1, TM7, *Cyanobacteria, Spirochaetes, Synergistetes*, and *Tenericutes*, are also found (<1% distribution). It is well established that oral-resident microbiota in poly-microbial interactions and soft-tissue biofilms avert oral diseases, but direct effect of such interactions on host oral immune cells is less clear (161–166). Oral mucosa maintains subsets of dendritic cells (DC), which produce immunomodulatory cytokines such as IL-10, TGF-β1 and Prostaglandin E2, and are predominantly tolerogenic (89, 167–169). These cells may be in intimate cross-talk with oral mucosal T_regs_ (58, 62, 170, 171), albeit details of such interactions between these cells are unexplored in oral mucosa. However, alterations in T_regs_ and Th17 functions have been implicated in human oral *Candida* infections and periodontitis (36, 38, 40, 69, 172–176). We and others have shown the presence of oral mucosal Foxp3^+^ T_regs_ with protective functions during local infection (89, 158, 169, 170). The interrelationship between these cells and oral commensals during an oral infection was also explored (58, 170). In the context of oropharyngeal candidiasis (OPC) infection, T_reg_ cells play a critical role in reducing fungal burden and establishing homeostasis during post anti-fungal response (177). T_regs_ play rather an unconventional role of enhancing the Th17 cell response and neutrophil infiltration during early acute response, but are associated with reduced TNF-α expression in CD4 T cells at resolution phase (83, 91, 178). *Candida* infection in mice by itself increases the proportion of Foxp3^+^T_regs_, in a TLR2/MyD88 dependent manner in oral mucosal tissues and draining cervical lymph nodes (58, 83, 91). A small proportion of those Foxp3^+^ cells co-express RORγt and IL-17A (T_reg_17). Antibiotic mediated depletion of resident bacteria significantly diminishes the frequency of Foxp3^+^T_reg_ IL-17A^−^ and T_reg_17 cells, as well as conventional Th17 cells not expressing Foxp3. Reduction of these cells is concomitant with an increase in tissue pathology and fungal burden in oral mucosa, demonstrating that resident bacteria are important for controlling Foxp3^+^ cells and Th17 cells, as well as mucosal immunity (Figure 1). Interestingly, *Candida* can also promote Th17 and T_reg_ responses in oral mucosa (83, 179, 180). The impact of oral resident microbiome in periodontal inflammation, which is now considered a “resident microbial perturbation” rather than a disease caused by a single pathogen, is well known (181). Resident bacterium *P. gingivalis*, the keystone pathogen contributes to altering the abundance and composition of other normal microbiota. Shift and accumulation of gram-positive aerobes to gram-negative anaerobes such as *P. gingivalis, T. denticola, F. nucleatum, and Prevotella sp*. are strongly associated with damage in gingival barrier, loss of immune balance and destruction of oral tissue in periodontal disease (150). During this process, bacterial antigens from skewed microbiota can access connective tissues causing abnormal activation and expansion of inflammatory CD4^+^CD69^+^CD103^−^ memory T cells and Th17 cells (182). Another recent study showed that periodontitis-associated expansion of Th17 cells required both IL-6 and IL-23, and was dependent on the local dysbiotic microbiome (31). Shift in resident microbiota can also include increase in *C. albicans*, a part of resident mycobiome in ~50–70% of healthy humans, which can rapidly transition to a pathogen and cause infections in immune-compromised and cancer patients. *C. albicans* is also shown to heighten *P. gingivalis* accumulation, worsening the series of inflammatory events associated with periodontitis severity (183, 184). It is known that T_reg_17 cells exist in periodontitis lesions and could be involved in inflammatory responses against periodontopathic bacteria (185). While there may be only small changes in oral microbiome in HIV+ individuals, underlying mechanisms causing dysbiosis and its association with HIV associated periodontitis during SIV/HIV infection are unclear (117, 186, 187). Precise events defining Th17 and T_reg_ dysfunctions in the context of underlying dysbiosis and aggravating oral inflammation in HIV disease and periodontitis remain to be seen.

## Microbiome in Mucosal Immunity and Inflammation in Other Mucosae

Lung, previously thought to be sterile, is now known to harbor a complex and dynamic microbial community of ~500 species, with a high resemblance to oral microbiome (188, 189). Lung microbiome strongly influences the development and progression of allergic responses and asthma (190). Disrupting the normal microbiome with childhood antibiotic exposure increases the risk of childhood asthma. *Proteobacteria* abundance in lower airway secretions correlates with pro-inflammatory Th17 cell proportions in asthmatic individuals (191, 192). Similarly, in cystic fibrosis patients, alterations of some groups in the polymicrobial community significantly affect the disease progression. Also, in chronic obstructive pulmonary disease (COPD) patients, microbial dysbiosis associated with mucus hyper-secretion and reduced airway clearance results in chronic aberrant inflammation and airway damage (193). Lung microbiota alterations are also associated with differences in pneumococcal clearance (194).

Multiple genera of microbiota exist in vaginal mucosa, often dominated by species of *Lactobacillus*, and a diverse array of anaerobic microorganisms, including *Atopobium, Anaerococcus, Corynebacterium, Eggerthella, Gardnerella, Mobiluncus, Peptoniphilus, Prevotella, Sneathia*, and *Finegoldia* genera (195). *Lactobacilli* largely impact the susceptibility to *T. vaginalis* infection in women. Although mechanisms are still under investigation, there is precedence that Th17 cells and T_regs_ can have protective and anti-inflammatory effects during *T. vaginalis* infection (196). During a vaginal herpes simplex virus-2 (HSV-2) infection, mice lacking T_regs_ fail to timely accumulate HSV-2-specific CD4 T cells and control the infection. This finding underscores the protective role of T_regs_ in facilitating productive mucosal immunity in vaginal mucosa (197, 198). However, mechanisms of direct control of vaginal microbiome on T_regs_ and Th17 cells and infection responses remain to be seen. In ocular mucosa, *Corynebacterium mastiditis* induces commensal specific IL-17 response γδ T cells, recruiting neutrophils and protecting the ocular mucosa from pathogenic infections (199). In nasal mucosa, on the one hand there is evidence that butyric acid-producing microorganisms associate with an impaired olfactory function (200–202). On the other, nasal microbiome is structured by IL-17 Signaling that that supports resistance to *S. pneumoniae* colonization in the nasal mucosa of mice (203). Collectively, while mcrobial dysbiosis and T_regs_/Th17 changes are associated with many of these infections, detailed mechanisms remain to be investigated.

## Molecular Mechanisms of Microbiota-Associated Alterations of T_REG_/Th17 Cells in Mucosae

**Figure 2:**
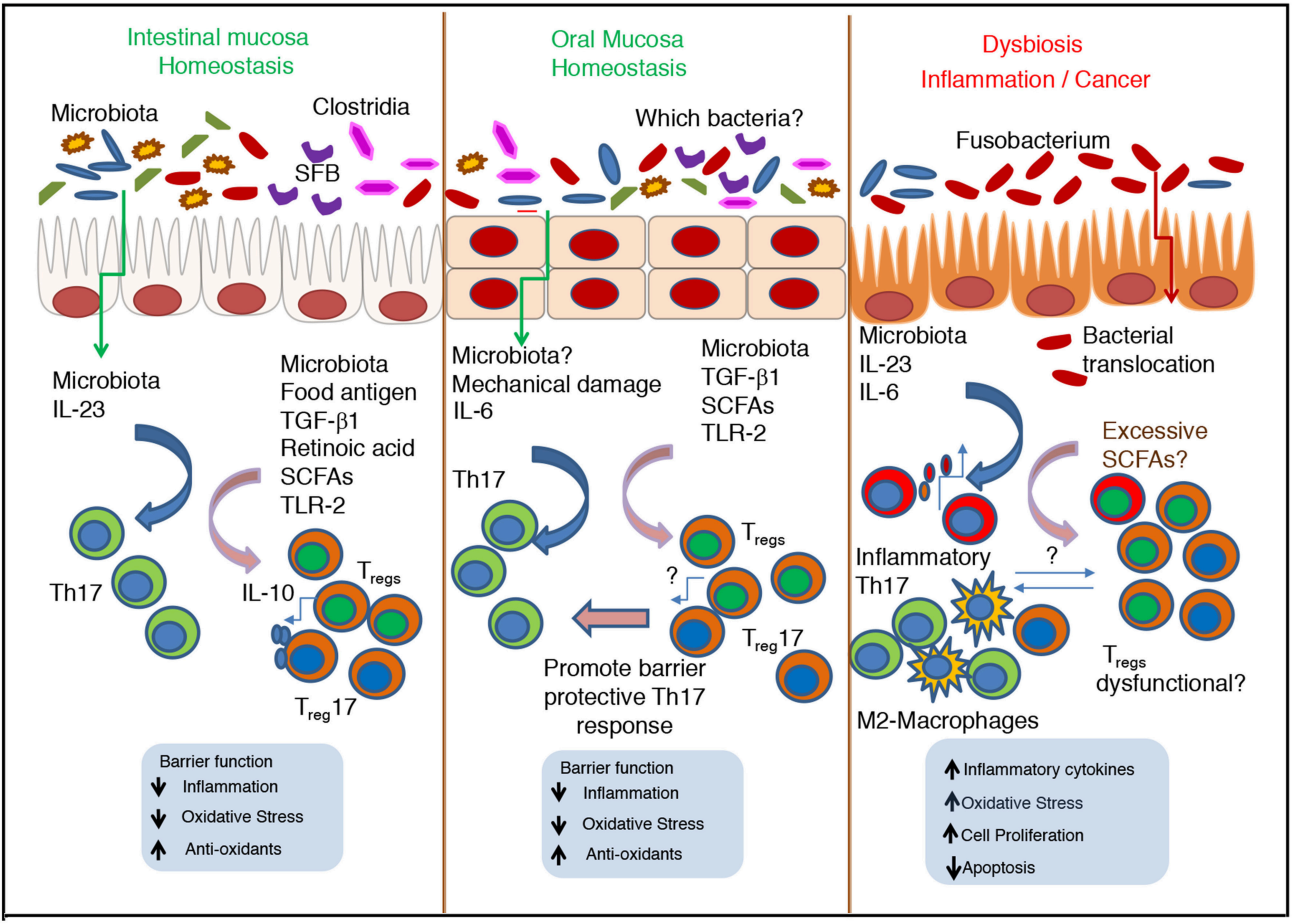
Cross talk between microbiota and immune cells during homeostasis and dysbiosis–Role of Th17 cells and T_regs_ in oral and intestinal mucosa. During homeostatic conditions, normal microbiota promote the stimulation of epithelial cells, Th17 cells and T_regs_, and maintain barrier function and commensal tolerance. In oral mucosa, Th17 cells are induced by mastication induced mechanical damage, independent of commensals. However, in both mucosae SCFA mediated induction of T_regs_ is key for mucosal barrier function and immunomodulation. During inflammation and cancer, excessive SCFAs can increase inflammatory Th17 cells and T_reg_ population that may be dysfunctional. The nature of their interaction with Th17 cells, tumor associated M2-type macrophages and other cells remain unclear.

Resident microbes have a variety of mechanisms for conferring mucosal colonization resistance (17, 204–207). They include: (1) directly competing for shared metabolites, (2) expression of inhibitory bacteriocins, (3) induction of protective mucus layer, and (4) priming of protective immune responses (208, 209). Some of the examples include commensal dependent metabolism of secondary bile acids to deoxycholate, production of organic acids, induction of antimicrobial peptides in Paneth cells, and promoting elevated antibacterial T cell responses preventing colonization and dissemination of pathogens (210–213). Although resident bacteria are known to modulate energy metabolism producing pyruvic acid, citric acid, fumaric acid and malic acid (214), how pH changes determine the mucosal immunity and T cells warrants further investigation. Resident microbiota employ multiple mechanisms that contribute to coordination of T_reg_/Th17 axis and safeguarding of mucosa (Figure 2). For example, microbiota dependent TLR signaling in host is one of the important mechanisms by which microbiota control inflammation and tolerance. TLR2/MyD88signaling is required for generation and expansion of Nrp1^low^ Foxp3+ cells and T_reg_17 cells in oral and gut mucosa (58). In gut mucosa the capsular polysaccharide A of the *Bacteroides fragilis* stimulates production of IL-10 by Foxp3^+^ cells in a TLR2 dependent manner, thus facilitating mucosal tolerance (215). Recently it was found that this commensal also delivers immunomodulatory molecules to immune cells via secretion of outer membrane vesicles through a non-canonical autophagy pathway for inducing IL-10 expressing Foxp3^+^ cells. This mechanism requires the expression of host genes *ATG16L1* and *NOD2*, whose polymorphisms are known to be associated with IBD (216). Selective deletion of *Atg16l1* in T cells in mice also results in loss of Foxp3^+^ T_reg_ cells and spontaneous intestinal inflammation characterized by aberrant Th2 responses. These data indicate microbiota-host interactions intimately involve the processes of autophagy and T_reg_ differentiation. Moreover, loss of MyD88-STAT3 signaling in T_regs_ causes loss of mucosal T_regs_ and impaired T follicular regulatory cell interactions, resulting in poor IL-21 and anti-microbial IgA responses (217). Failure of this pathway results in over-growth of pathobionts, overt Th17 cell expansion and intestinal inflammation. However, the requirement of resident microbiome induced MyD88 signaling specifically in T_regs_, to promote T_reg_ sustenance and intestinal tolerance is still debated (217–219). Similar to *B.fragilis*, colonic *Clostridium rhamnosus* also potently induces IL-10^+^T_regs_ in a TGF-β1 dependent manner, which is correlated to increase in systemic IgE and resistance to colonic inflammation (56, 99). Similarly, microbiota and immune cell networks are known to control the production of IgA, which is central for mucosal barrier and intestinal tolerance. For example, *Mucispirillum* spp. and SFB have been directly implicated in production of intestinal IgA (137, 220, 221). T_regs_ are also known to promote IgA secretion, and maintenance of diversified and balanced microbiota, which in turn facilitates their expansion through a symbiotic regulatory loop, and prevent overt inflammation (222, 223). Moreover, RORγt^+^ Th17 cells, as well as IL-17A from other cells also promote epithelial polymeric Ig receptor and intestinal IgA expression, further contributing to intestinal homeostasis (224, 225). SFB also control commensal tolerance and anti-microbial host responses through intestinal epithelial cell fucosyl tranferase 2 expression and fucosylation, a process that is dependent on RORγt^+^ group 3 innate lymphoid cells (ILC3s) and IL-22 expression (226, 227). Loss of intestinal fucosylation results in increased susceptibility to infection by *Salmonella typhimurium*. ILC3s can also express major histocompatibility complex class II (MHCII) and mediate intestinal selection of CD4^+^ T cells in order to limit commensal bacteria-specific CD4 T-cell responses (228). Although IL-6, induction of T_regs_, or Th17 cells were shown to be not required for ILC-mediated tolerance, alterations in T_reg_17 and Th17 cells in the context of fucosylation remain to be studied. T_reg_/Th17 cell differentiation and expansion are also independently controlled by specific members of anaerobic bacteria producing short chain fatty acids (SCFAs), such as acetate, propionate and butyrate (229, 230). Some of these bacteria include *Bacteroides, Bifidobacterium, Feacalibacterium* genera, *and Enterobacteriaceae family, Porphyromonas gingivalis, Fusobacterium nucleatum* (mouth), *Clostridium cochlearium, Eubacterium multiforme* (intestine), and *Anaerococcus tetradius* (vagina). These bacteria ferment indigestible oligosaccharides and cell surface fucosylated proteins by anaerobic glycolysis, resulting in SCFA production. SCFAs are present in the intestinal lumen at a total concentration of ~100 mM at a ratio of ~6:3:1, for acetate, propionate and butyrate respectively. Although this ratio hinges on carbohydrate availability, microbiota composition and intestinal transit time, acetate and butyrate appear to be the highest and least in abundance respectively (231). Emerging data show that SCFAs contribute to immune homeostasis in mucosa, although excessive and suboptimal levels of SCFAs are often associated with inflammation and cancer. Intestinal SCFAs have been shown to potentiate Foxp3^+^ cell differentiation and immunomodulatory activity in the colon (53, 99, 147, 232). Mechanistically, in addition to direct histone deacetylase (HDAC) inhibition, SCFAs can induce the expression of retinal aldehyde dehydrogenase 1 family member 1a (Aldh1a) and TGF-β1 in intestinal epithelial cells and DCs (100, 221, 233, 234). Aldh1a could further convert vitamin A into its metabolite retinoic acid in G protein–coupled receptor43 (GPCR43) and Gpr109a manner, which is capable of facilitating T_reg_ induction. These tolerogenic DCs express CD103, sample antigens in the intestinal lamina propria, and migrate to the draining mesenteric lymph node (MLN) to induce immunomodulatory T cells (235–237). Whether SCFA mediated induction and or sustenance of mucosal T_regs_ require these aforementioned processes is unclear and remain to be studied. However, antibiotics precipitously decrease the oral SCFAs in saliva, showing that in the oral resident bacteria-derived-SCFA is functionally involved in controlling oral mucosal immunity and inflammation (62). Lending credence to this tenet, antibiotics treated mice show not only increased oral inflammation, but also intestinal immunopathology, when infected with oral *Candida*. Mechanistically, antibiotic treatment results in reduced T_regs_, Th17 and T_reg_17 cells in oral mucosa and tissue draining cervical and axillary lymph nodes in infected mice. Intestinal inflammation in oral *Candida* infected mice is characterized by an increase in IFN-γ producing Th1 cells and co-producers of IFN-γ and IL-17A (Th1^*^) cells. Although the exact mechanism of antibiotic mediated reduction of T_regs_, Th17 cells and T_reg_17 cells is unclear, administration of SCFA partially restored these populations and reduces oral immunopathology during the infection. SCFA administration however, only moderately ameliorates the intestinal inflammation. Therefore, the mechanism of Th1-mediated gut inflammation during oral *Candida* infection in the context of altered microbiota remains to be addressed. Recently, Atarashi et al. showed that oral bacterium *Klebsiella* spp. isolated from the salivary microbiota elicits a severe Th1 gut inflammation in the context of intestinal dysbiosis, in a genetically susceptible host (238). This finding underscores the role of oral resident microbes such as *Klebsiella* spp. and *C. albicans* in modulating T cells, possibly translocating to gut and causing overt inflammation in the gut in the context of resident microbial dysbiosis. Supporting this tenet, post oral gavage of *C. albicans*-infected mice pre-treated with antibiotics showed significantly altered composition of intestinal microbiota as well as CD4^+^ T cell mediated lung inflammation, following aerosol introduction of an allergen. However, mice without any antibiotics pre-treatment did not develop an allergic response in the airways (239, 240). Whether changes in SCFA, or T_reg_ and Th17 cells in the lung contribute to the inflammation is unknown.

Mechanistically, SCFAs also cause acetylation of p70 S6 kinase and phosphorylation rS6, promoting the mTOR activity. mTOR activity was shown to be required for generation of Th17 (T helper type 17), Th1, and IL-10^+^ T cells (241). Moreover phosphoinositide 3-kinase and mTOR pathways play pivotal roles in integrating growth signals in CD4^+^ T cell differentiation (242–249). Multiple studies support the role of mTORC1 and mTORC2 proteins in regulating Th17 and T_reg_ fate decisions (247, 250, 251). mTORC1 signaling is constitutively active in T_reg_ cells, and disruption of mTOR protein as well as unrestrained mTOR hyper-activation, both have been shown to cause autoimmunity by impairing Foxp3 expression and T_reg_ functions (252–260). Another study has also shown that mTORC1 and its downstream target hypoxia-inducible factor-1α (HIF-1α) are needed for Foxp3 induction, T_reg_ lipid and cholesterol biosynthesis from glucose, and proliferation and suppressive function *in vivo* (244, 254). Taken together, while direct role of SCFA in mediating mTOR activation and subsequent T_reg_ induction in mucosa is unclear, these studies highlight the importance of how immunologically relevant microbiome can control T_regs_ and mucosal homeostasis through multiple mechanisms.

## Microbiota and T_REG_/Th17 Cell Regulation of Immune Senescence and Chronic Inflammation

While resident microbes have aforementioned protective functions in mucosa, they can also trigger and sustain inflammation during aging and other chronic inflammatory conditions. Some studies demonstrate direct relationship between aging and changes in microbiota, albeit the mechanisms remain largely unstudied. Aging causes increased accumulation of gut *Enterobacteria, Streptococci, and yeasts* but declining levels of *Akkermansia muciniphila, Bifidobacteria* and *Bacteroides* (261–266). Reduced *Akkermansia muciniphila* is associated with reduced butyrate and impaired intestinal barrier. Consequently, aged mice display endotoxin leakage, and triggering of 4-1BB receptor signaling and insulin resistance. In oral mucosa, aging causes higher levels of RANKL^+^ cells, and increased inflammatory Th17 cell accumulation, with concomitant loss of alveolar bone, which are dependent on the presence of commensal microbiota (30, 267, 268). In contrast, these events do not occur in in germfree mice periodontium, showing potentially pathogenic roles of commensal microbiota in aging associated dysbiosis setting. Similarly, resident microbiota have been implicated in the onset and progression of experimental autoimmune encephalomyelitis (EAE) (269). GF mice exhibit lower levels of the pro-inflammatory cytokine IFN-γ and IL-17A producing cells, and a reciprocal increase in T_regs_ in the intestine and spinal cord. These changes in GF mice correlate with a significantly attenuated EAE, compared with conventionally raised mice. Remarkably, intestinal colonization with SFB alone can promote Th17 cells in the gut and in the central nervous system (CNS), enhancing disease progression (270). Furthermore, partial elimination of intestinal microbiota ameliorates established collagen-induced arthritis by dampening Th17 responses in mice (271). Some bacteria also provide inflammatory signals resulting in chronic inflammation and tumorigenesis, likely by inducing genetic and epigenetic changes in host cells. For example, *Fusobabacteria* spp. has been implicated in increased risk of IBD and colorectal cancer (272–275). Also, in oral mucosa, the abundance of *Fusobacterium* increases, while the number of *Streptococcus, Haemophilus, Porphyromonas, and Actinomyces* decreases with cancer progression in oral squamous cell carcinoma (276). Interestingly, *Fusobabacteria*, and several other bacteria of oral mucosal origin, including genera of *Streptococcus, Staphylococcus, Peptostreptococcus* may translocate to intestine in the context of gut inflammation and carcinogenesis (277–279), similar to *Klebsiella* spp *and C. albicans* in susceptible host (62, 238). It is tempting to speculate that loss of T_reg_ functions in the context of dybiosis, excessive SCFA and oral microbial translocation may have contributed to exuberant intestinal inflammation and predisposition to carcinogenesis in these studies (Figure 2). However, whether the mouth- to -gut translocation is a cause, or consequence of dysbiosis and intestinal inflammation, and the underlying mechanisms still remain to be understood and warrant further investigation.

## Therapeutic Applications of Microbiota Alterations and Microbiota Derived Metabolites.

As we discussed above, studies on patient cohorts, mechanistic studies on mice and epidemiological studies have led to a better understanding of how microbiota changes impact mucosal immunity, and *vice versa*. Mechanistic “proof-of principle” studies using disease models have opened ways to manipulate these processes, providing therapeutic approaches. Some of the widely used approaches include administration of sodium butyrate and pre- and pro-biotics, and transplantation of fecal microbiota (280–283). However, there are hurdles in pro-biotic and microbiota transplantation approaches. Existing microbiota, whether it is healthy or dysbiotic is largely stable over time in an individual. Without profound perturbation of the existing microbiota, it is challenging to introduce microbiota exogenously. The effects of exogenous bacteria introduced by probiotic and transplant approaches are greatly influenced by existing microbiota in a competitive niche, and are inconsistent. Therefore, approaches to target these niches in favor of exogenous bacteria are being studied (283, 284). Direct administration of microbial derivatives appears to be a promising venue. Butyrate has been shown to alleviate high-fat-diet induced non-alcoholic fatty liver disease. It potently down modulates peroxisome proliferator-activated receptor α-mediated activation of β oxidation, causing reduced inflammation (285). For cART treated HIV^+^ individuals, aside from cART treatment, probiotics have been studied to combat persistent systemic inflammation. This approach in the context of cART may lead to improved and holistic management of inflammatory events and higher cancer susceptibility in HIV+ patients. Application of probiotics has also shown positive effect on the course of pneumonia, acute exacerbation of bronchial asthma and COPD in mice models, but warrants further studies in humans (286). SCFA has been shown to have therapeutic potential in microbiome-targeted interventions in anti-aging medicine. Butyrate and dietary fibers have been shown to promote anti-inflammatory effects in the context of aging associated neuro-inflammation in mice (287). Adult and aged mice fed with 5% inulin (high fiber) diet for 4 weeks show an altered gut microbiome and increased butyrate, acetate, and total SCFA production, coinciding with a reduction in neuro-inflammation. High fiber supplementation in aging is a non-invasive strategy to increase butyrate levels, and these data suggest that an increase in butyrate through added soluble fiber such as inulin could counterbalance the age-related microbiota dysbiosis, potentially leading to neurological benefits (287, 288). Similarly, dietary fiber also suppresses colon carcinogenesis in polyposis mice (289). Mechanistically it has been shown to inhibit colorectal cancer cell migration through micro-RNA regulation (290). In summary, alterations of mechanisms of microbiota-host interactions are proving to hold promise for treating a variety of disorders in humans.

## Conclusion

It is now well established that resident microbes provide enormous advantages to the host, while dysbiosis can trigger acute and chronic inflammatory conditions. One of the mechanisms by which these microbes regulate immunity id through controlling T_regs_ and Th17 cells. These cells present in various mucosal locations and share various signaling pathways for their development and sustenance, as stated above. However, signals modulating these subsets unique to each mucosal environment in different epithelial cell contexts are unclear. Most mechanistic studies showing T_reg_/Th17 developmental regulation were performed using the *in vitro* cultures using cells isolated from blood (human), spleen and lymph nodes (mice). While there is enough evidence to show that these cells could be regulated by overlapping signaling mechanisms, cells from these mucosae were not directly compared for similarities and differences in their development and functions. Such studies are warranted to get further insights in to homeostatic and dysbiotic conditions in different mucosae. Such studies in the context of microbial manipulation approach will offer new avenues to manipulate their interactions with the host for treating immune-mediated and metabolic disorders. While mono-association of certain genera in GF mice have proven to alter mucosal T_regs_ and Th17 cells and offer some beneficial effects in some experimental settings (98), from a therapeutic perspective, the field is still at its infancy and warrants intense mechanistic investigations. Taken together, further research in microbiota targeted approaches will enable the field to take the center stage in the management of health and disease in humans.

## Author Contributions

PP and JH wrote the manuscript. NB, ES, MZ, and SJ contributed to the discussion.

### Conflict of Interest Statement

The authors declare that the research was conducted in the absence of any commercial or financial relationships that could be construed as a potential conflict of interest.

## References

[1] Lloyd-Price J Abu-Ali G Huttenhower C. The healthy human microbiome. Genome Med. (2016) 8:51. 10.1186/s13073-016-0307-y 27122046 PMC4848870

[2] Savage DC. Microbial ecology of the gastrointestinal tract. Annu Rev Microbiol. (1977) 31:107–33. 10.1146/annurev.mi.31.100177.000543 334036

[3] Donaldson GP Lee SM Mazmanian SK. Gut biogeography of the bacterial microbiota. Nat Rev Microbiol. (2016) 14:20–32. 10.1038/nrmicro3552 26499895 PMC4837114

[4] Sender R Fuchs S Milo R. Revised estimates for the number of human and bacteria cells in the body. PLoS Biol. (2016) 14:e1002533. 10.1371/journal.pbio.1002533 27541692 PMC4991899

[5] Gallo RL Nakatsuji T. Microbial symbiosis with the innate immune defense system of the skin. J Invest Dermatol. (2011) 131:1974–80. 10.1038/jid.2011.182 21697881 PMC3174284

[6] Abt MC Osborne LC Monticelli LA Doering TA Alenghat T Sonnenberg GF. Commensal bacteria calibrate the activation threshold of innate antiviral immunity. Immunity. (2012) 37:158–70. 10.1016/j.immuni.2012.04.011 22705104 PMC3679670

[7] Flint HJ Scott KP Louis P Duncan SH. The role of the gut microbiota in nutrition and health. Nat Rev Gastroenterol Hepatol. (2012) 9:577–89. 10.1038/nrgastro.2012.156 22945443

[8] Hill DA Siracusa MC Abt MC Kim BS Kobuley D Kubo M. Commensal bacteria-derived signals regulate basophil hematopoiesis and allergic inflammation. Nat Med. (2012) 18:538–46. 10.1038/nm.2657 22447074 PMC3321082

[9] Abt MC Artis D. The dynamic influence of commensal bacteria on the immune response to pathogens. Curr Opin Microbiol. (2013) 16:4–9. 10.1016/j.mib.2012.12.002 23332724 PMC3622187

[10] Hill DA Artis D. The influence of commensal bacteria-derived signals on basophil-associated allergic inflammation. Gut Microb. (2013) 4:76–83. 10.4161/gmic.22759 23137965 PMC3555891

[11] Kamada N Chen GY Inohara N Nunez G. Control of pathogens and pathobionts by the gut microbiota. Nat Immunol. (2013) 14:685–90. 10.1038/ni.2608 23778796 PMC4083503

[12] Kamada N Seo SU Chen GY Nunez G. Role of the gut microbiota in immunity and inflammatory disease. Nat Rev Immunol. (2013) 13:321–35. 10.1038/nri3430 23618829

[13] Spasova DS Surh CD. Blowing on embers: commensal microbiota and our immune system. Front Immunol. (2014) 5:318. 10.3389/fimmu.2014.00318 25120539 PMC4112811

[14] Macpherson AJ Harris NL. Interactions between commensal intestinal bacteria and the immune system. Nat Rev Immunol. (2004) 4:478–85. 10.1038/nri1373 15173836

[15] Karin M Lawrence T Nizet V. Innate immunity gone awry: linking microbial infections to chronic inflammation and cancer. Cell. (2006) 124:823–35. 10.1016/j.cell.2006.02.016 16497591

[16] Lee YK Mazmanian SK. Has the microbiota played a critical role in the evolution of the adaptive immune system? Science. (2010) 330:1768–73. 10.1126/science.1195568 21205662 PMC3159383

[17] Belkaid Y Naik S. Compartmentalized and systemic control of tissue immunity by commensals. Nat Immunol. (2013) 14:646–53. 10.1038/ni.2604 23778791 PMC3845005

[18] Brito F Zaltman C Carvalho AT Fischer RG Persson R Gustafsson A. Subgingival microflora in inflammatory bowel disease patients with untreated periodontitis. Eur J Gastroenterol Hepatol. (2013) 25:239–45. 10.1097/MEG.0b013e32835a2b70 23060013

[19] Huffnagle GB Noverr MC. The emerging world of the fungal microbiome. Trends Microbiol. (2013) 21:334–41. 10.1016/j.tim.2013.04.002 23685069 PMC3708484

[20] Belkaid Y Hand TW. Role of the microbiota in immunity and inflammation. Cell. (2014) 157:121–41. 10.1016/j.cell.2014.03.011 24679531 PMC4056765

[21] Blander JM Longman RS Iliev ID Sonnenberg GF Artis D. Regulation of inflammation by microbiota interactions with the host. Nat Immunol. (2017) 18:851–60. 10.1038/ni.3780 28722709 PMC5800875

[22] Bilate AM Bousbaine D Mesin L Agudelo M Leube J Kratzert A. (2016). Tissue-specific emergence of regulatory and intraepithelial T cells from a clonal T cell precursor. Sci Immunol 1:eaaf7471. 10.1126/sciimmunol.aaf7471 28783695 PMC6296461

[23] Calderon-Gomez E Bassolas-Molina H Mora-Buch R Dotti I Planell N Esteller M. Commensal-Specific CD4(+) Cells From Patients With Crohn's Disease Have a T-Helper 17 Inflammatory Profile. Gastroenterology. (2016) 151:489–500 e483. 10.1053/j.gastro.2016.05.050 27267052

[24] Hegazy AN West NR Stubbington MJT Wendt E Suijker KIM Datsi A. Circulating and tissue-resident CD4(+) T cells with reactivity to intestinal microbiota are abundant in healthy individuals and function is altered during inflammation. Gastroenterology. (2017) 153:1320–37 e1316. 10.1053/j.gastro.2017.07.047 28782508 PMC5687320

[25] Navabi N Whitt J Wu SE Woo V Moncivaiz J Jordan MB. Epithelial histone deacetylase 3 instructs intestinal immunity by coordinating local lymphocyte activation. Cell Rep. (2017) 19:1165–75. 10.1016/j.celrep.2017.04.046 28494866 PMC5499685

[26] Sonnenburg ED Smits SA Tikhonov M Higginbottom SK Wingreen NS Sonnenburg JL. Diet-induced extinctions in the gut microbiota compound over generations. Nature. (2016) 529:212–5. 10.1038/nature16504 26762459 PMC4850918

[27] Stappenbeck TS Virgin HW. Accounting for reciprocal host-microbiome interactions in experimental science. Nature. (2016) 534:191–9. 10.1038/nature18285 27279212

[28] Willyard C. Squeaky clean mice could be ruining research. Nature. (2018) 556:16–8. 10.1038/d41586-018-03916-9 29620765

[29] Kim KS Hong SW Han D Yi J Jung J Yang BG. Dietary antigens limit mucosal immunity by inducing regulatory T cells in the small intestine. Science. (2016) 351:858–63. 10.1126/science.aac5560 26822607

[30] Dutzan N Abusleme L Bridgeman H Greenwell-Wild T Zangerle-Murray T Fife ME. On-going mechanical damage from mastication drives homeostatic Th17 cell responses at the oral barrier. Immunity. (2017) 46:133–47. 10.1016/j.immuni.2016.12.010 28087239 PMC5263257

[31] Dutzan N Kajikawa T Abusleme L Greenwell-Wild T Zuazo CE Ikeuchi T. A dysbiotic microbiome triggers TH17 cells to mediate oral mucosal immunopathology in mice and humans. Sci Transl Med. (2018) 10:eaat0797. 10.1126/scitranslmed.aat0797 30333238 PMC6330016

[32] Klein RS Harris CA Small CB Moll B Lesser M Friedland GH. Oral candidiasis in high-risk patients as the initial manifestation of the acquired immunodeficiency syndrome. N Engl J Med. (1984) 311:354–8. 10.1056/NEJM198408093110602 6738653

[33] Patton LL. Sensitivity, specificity, and positive predictive value of oral opportunistic infections in adults with HIV/AIDS as markers of immune suppression and viral burden. Oral Surg Oral Med Oral Pathol Oral Radiol Endod. (2000) 90:182–8. 10.1067/moe.2000.108799 10936837

[34] Patton LL McKaig R Strauss R Rogers D Eron JJ Jr. Changing prevalence of oral manifestations of human immuno-deficiency virus in the era of protease inhibitor therapy. Oral Surg Oral Med Oral Pathol Oral Radiol Endod. (2000) 89:299–304. 10.1016/S1079-2104(00)70092-8 10710453

[35] Gaitan Cepeda LA Ceballos Salobrena A Lopez Ortega K Arzate Mora N Jimenez Soriano Y. Oral lesions and immune reconstitution syndrome in HIV+/AIDS patients receiving highly active antiretroviral therapy. Epidemiological evidence. Med Oral Patol Oral Cir Bucal. (2008) 13:E85–93. 18223535

[36] Kanwar B Favre D McCune JM. Th17 and regulatory T cells: implications for AIDS pathogenesis. Curr Opin HIV AIDS. (2010) 5:151–7. 10.1097/COH.0b013e328335c0c1 20543593 PMC2999911

[37] Fidel PL Jr. (2011). *Candida*-host interactions in HIV disease: implications for oropharyngeal candidiasis. Adv Dent Res. 23, 45–49. 10.1177/0022034511399284 21441480 PMC3144040

[38] Li D Chen J Jia M Hong K Ruan Y Liang H. Loss of balance between T helper type 17 and regulatory T cells in chronic human immunodeficiency virus infection. Clin Exp Immunol. (2011) 165:363–71. 10.1111/j.1365-2249.2011.04435.x 21707592 PMC3170985

[39] Cassone A Cauda R. *Candida* and candidiasis in HIV-infected patients: where commensalism, opportunistic behavior and frank pathogenicity lose their borders. AIDS. (2012) 26:1457–72. 10.1097/QAD.0b013e3283536ba8 22472853

[40] Huppler AR Bishu S Gaffen SL. Mucocutaneous candidiasis: the IL-17 pathway and implications for targeted immunotherapy. Arthr Res Ther. (2012) 14:217. 10.1186/ar3893 22838497 PMC3580547

[41] Pandiyan P Younes S Ribeiro S Talla A Bhaskaran N McDonald D. Mucosal regulatory T cells and T helper 17 cells in HIV associated immune activation. Front immunol. (2016) 7:228. 10.3389/fimmu.2016.00228 27379092 PMC4913236

[42] Pandiyan P Zheng L Ishihara S Reed J Lenardo MJ. CD4(+)CD25(+)Foxp3(+) regulatory T cells induce cytokine deprivation-mediated apoptosis of effector CD4(+) T cells. Nat Immunol. (2007) 8:1353–62. 10.1038/ni1536 17982458

[43] Pandiyan P Zheng L Lenardo MJ. The molecular mechanisms of regulatory T cell immunosuppression. Front Immunol. (2011) 2:60. 10.3389/fimmu.2011.00060 22566849 PMC3342245

[44] Burzyn D Kuswanto W Kolodin D Shadrach JL Cerletti M Jang Y. A special population of regulatory T cells potentiates muscle repair. Cell. (2013) 155:1282–95. 10.1016/j.cell.2013.10.054 24315098 PMC3894749

[45] Edwards JP Fujii H Zhou AX Creemers J Unutmaz D Shevach EM. Regulation of the expression of GARP/latent TGF-beta1 complexes on mouse T cells and their role in regulatory T cell and Th17 differentiation. J Immunol. (2013) 190:5506–15. 10.4049/jimmunol.1300199 23645881 PMC3668701

[46] Arpaia N Green JA Moltedo B Arvey A Hemmers S Yuan S. A Distinct Function of Regulatory T *Cell*s in Tissue Protection. Cell. (2015) 162:1078–89. 10.1016/j.cell.2015.08.021 26317471 PMC4603556

[47] Worthington JJ Kelly A Smedley C Bauche D Campbell S Marie JC. Integrin alphavbeta8-Mediated TGF-beta Activation by Effector Regulatory T Cells Is Essential for Suppression of T-Cell-Mediated Inflammation. Immunity. (2015) 42:903–15. 10.1016/j.immuni.2015.04.012 25979421 PMC4448149

[48] Jin RM Warunek J Wohlfert EA. Therapeutic administration of IL-10 and amphiregulin alleviates chronic skeletal muscle inflammation and damage induced by infection. Immunohorizons. (2018) 2:142–54. 10.4049/immunohorizons.1800024 30417170 PMC6223302

[49] Povoleri GAM Nova-Lamperti E Scotta C Fanelli G Chen YC Becker PD. Human retinoic acid-regulated CD161(+) regulatory T cells support wound repair in intestinal mucosa. Nat Immunol. (2018) 19:1403–14. 10.1038/s41590-018-0230-z 30397350 PMC6474659

[50] Izcue A Coombes JL Powrie F. Regulatory lymphocytes and intestinal inflammation. Annu Rev Immunol. (2009) 27:313–38. 10.1146/annurev.immunol.021908.132657 19302043

[51] Cong Y Feng T Fujihashi K Schoeb TR Elson CO. A dominant, coordinated T regulatory cell-IgA response to the intestinal microbiota. Proc Natl Acad Sci USA. (2009) 106:19256–61. 10.1073/pnas.0812681106 19889972 PMC2780781

[52] Cebula A Seweryn M Rempala GA Pabla SS McIndoe RA Denning TL. Thymus-derived regulatory T cells contribute to tolerance to commensal microbiota. Nature. (2013) 497:258–62. 10.1038/nature12079 23624374 PMC3711137

[53] Smith PM Howitt MR Panikov N Michaud M Gallini CA Bohlooly YM. The microbial metabolites, short-chain fatty acids, regulate colonic Treg cell homeostasis. Science. (2013) 341:569–73. 10.1126/science.1241165 23828891 PMC3807819

[54] Lathrop SK Bloom SM Rao SM Nutsch K Lio CW Santacruz N. Peripheral education of the immune system by colonic commensal microbiota. Nature. (2011) 478:250–4. 10.1038/nature10434 21937990 PMC3192908

[55] Nutsch K Chai JN Ai TL Russler-Germain E Feehley T Nagler CR. Rapid and efficient generation of regulatory T cells to commensal antigens in the periphery. Cell Rep. (2016) 17:206–20. 10.1016/j.celrep.2016.08.092 27681432 PMC5051580

[56] Atarashi K Tanoue T Shima T Imaoka A Kuwahara T Momose Y. Induction of colonic regulatory T cells by indigenous *Clostridium* species. Science. (2011) 331:337–41. 10.1126/science.1198469 21205640 PMC3969237

[57] Geuking MB Cahenzli J Lawson MA Ng DC Slack E Hapfelmeier S. Intestinal bacterial colonization induces mutualistic regulatory T cell responses. Immunity. (2011) 34:794–806. 10.1016/j.immuni.2011.03.021 21596591

[58] Bhaskaran N Cohen S Zhang Y Weinberg A Pandiyan P. TLR-2 signaling promotes IL-17A production in CD4^+^CD25^+^Foxp3+ regulatory cells during oropharyngeal candidiasis. Pathogens. (2015) 4:90–110. 10.3390/pathogens4010090 25790134 PMC4384074

[59] Hadis U Wahl B Schulz O Hardtke-Wolenski M Schippers A Wagner N. Intestinal tolerance requires gut homing and expansion of FoxP3+ regulatory T cells in the lamina propria. Immunity. (2011) 34:237–46. 10.1016/j.immuni.2011.01.016 21333554

[60] Weiss JM Bilate AM Gobert M Ding Y Curotto de Lafaille MA Parkhurst CN Neuropilin 1 is expressed on thymus-derived natural regulatory T cells, but not mucosa-generated induced Foxp3+ T reg cells. J Exp Med. (2012) 209:1723–42, S1721. 10.1084/jem.20120914 22966001 PMC3457733

[61] Yadav M Louvet C Davini D Gardner JM Martinez-Llordella M Bailey-Bucktrout S. Neuropilin-1 distinguishes natural and inducible regulatory T cells among regulatory T cell subsets *in vivo*. J Exp Med. (2012) 209:1713–22, S1711-9. 10.1084/jem.20120822 22966003 PMC3457729

[62] Bhaskaran N Quigley C Paw C Butala S Schneider E Pandiyan P. Role of short chain fatty acids in controlling tregs and immunopathology during mucosal infection. Front Microbiol. (2018) 9:1995. 10.3389/fmicb.2018.01995 30197637 PMC6117408

[63] Sakaguchi S The origin of FOXP3-expressing CD4^+^ regulatory T cells: thymus or periphery. J Clin Invest. (2003) 112:1310–2. 10.1172/JCI200320274 14597756 PMC228490

[64] Sakaguchi S Yamaguchi T Nomura T Ono M. Regulatory T cells and immune tolerance. Cell. (2008) 133:775–87. 10.1016/j.cell.2008.05.009 18510923

[65] Shevach EM. Mechanisms of foxp3+ T regulatory cell-mediated suppression. Immunity. (2009) 30:636–45. 10.1016/j.immuni.2009.04.010 19464986

[66] Panduro M Benoist C Mathis D. Tissue Tregs. Annu Rev Immunol. (2016) 34:609–33. 10.1146/annurev-immunol-032712-095948 27168246 PMC4942112

[67] Rowe JH Ertelt JM Aguilera MN Farrar MA Way SS. Foxp3(+) regulatory T cell expansion required for sustaining pregnancy compromises host defense against prenatal bacterial pathogens. Cell Host Microbe. (2011) 10:54–64. 10.1016/j.chom.2011.06.005 21767812 PMC3140139

[68] Johanns TM Ertelt JM Rowe JH Way SS. Regulatory T cell suppressive potency dictates the balance between bacterial proliferation and clearance during persistent Salmonella infection. PLoS Pathog. (2010) 6:e1001043. 10.1371/journal.ppat.1001043 20714351 PMC2920851

[69] Garlet GP Cardoso CR Mariano FS Claudino M de Assis GF Campanelli AP. Regulatory T cells attenuate experimental periodontitis progression in mice. J Clin Periodontol. (2010) 37:591–600. 10.1111/j.1600-051X.2010.01586.x 20642629

[70] Zhong Y Cantwell A Dube PH. Transforming growth factor beta and CD25 are important for controlling systemic dissemination following *Yersinia enterocolitica* infection of the gut. Infect Immun. (2010) 78:3716–25. 10.1128/IAI.00203-10 20584975 PMC2937473

[71] Eggena MP Barugahare B Jones N Okello M Mutalya S Kityo C. Depletion of regulatory T cells in HIV infection is associated with immune activation. J Immunol. (2005) 174:4407–14. 15778406 10.4049/jimmunol.174.7.4407

[72] Chase AJ Sedaghat AR German JR Gama L Zink MC Clements JE Severe depletion of CD4^+^ CD25+ regulatory T cells from the intestinal lamina propria but not peripheral blood or lymph nodes during acute simian immunodeficiency virus infection. J Virol. (2007) 81:12748–57. 10.1128/JVI.00841-07 17855517 PMC2169083

[73] Chase AJ Yang HC Zhang H Blankson JN Siliciano RF. Preservation of FoxP3+ regulatory T cells in the peripheral blood of human immunodeficiency virus type 1-infected elite suppressors correlates with low CD4^+^ T-cell activation. J Virol. (2008) 82:8307–15. 10.1128/JVI.00520-08 18579608 PMC2519624

[74] Elahi S Dinges WL Lejarcegui N Laing KJ Collier AC Koelle DM. Protective HIV-specific CD8+ T cells evade Treg cell suppression. Nat Med. (2011) 17:989–95. 10.1038/nm.2422 21765403 PMC3324980

[75] Lanteri MC O'Brien KM Purtha WE Cameron MJ Lund JM Owen RE. Tregs control the development of symptomatic West Nile virus infection in humans and mice. J Clin Invest. (2009) 119:3266–77. 10.1172/JCI39387 19855131 PMC2769173

[76] Oldenhove G Bouladoux N Wohlfert EA Hall JA Chou D Dos Santos L. Decrease of Foxp3^+^ Treg cell number and acquisition of effector cell phenotype during lethal infection. Immunity. (2009) 31:772–86. 10.1016/j.immuni.2009.10.001 19896394 PMC2814877

[77] Morampudi V De Craeye S Le Moine A Detienne S Braun MY D'Souza S. Partial depletion of CD4(+)CD25(+)Foxp3(+) T regulatory cells significantly increases morbidity during acute phase Toxoplasma gondii infection in resistant BALB/c mice. Microbes Infect. (2011) 13:394–404. 10.1016/j.micinf.2011.01.006 21262371

[78] Tenorio EP Olguin JE Fernandez J Vieyra P Saavedra R. Reduction of Foxp3+ cells by depletion with the PC61 mAb induces mortality in resistant BALB/c mice infected with Toxoplasma gondii. J Biomed Biotechnol. (2010) 2010:786078. 10.1155/2010/786078 20037737 PMC2796377

[79] Rausch S Huehn J Loddenkemper C Hepworth MR Klotz C Sparwasser T. Establishment of nematode infection despite increased Th2 responses and immunopathology after selective depletion of Foxp3+ cells. Eur J Immunol. (2009) 39:3066–77. 10.1002/eji.200939644 19750483

[80] Mendez S Reckling SK Piccirillo CA Sacks D Belkaid Y. Role for CD4(+) CD25(+) regulatory T cells in reactivation of persistent leishmaniasis and control of concomitant immunity. J Exp Med. (2004) 200:201–10. 10.1084/jem.20040298 15263027 PMC2212012

[81] Baumgart M Tompkins F Leng J Hesse M. Naturally occurring CD4^+^Foxp3+ regulatory T cells are an essential, IL-10-independent part of the immunoregulatory network in Schistosoma mansoni egg-induced inflammation. J Immunol. (2006) 176:5374–87. 10.4049/jimmunol.176.9.5374 16622005

[82] Montagnoli C Bacci A Bozza S Gaziano R Mosci P Sharpe AH. B7/CD28-dependent CD4^+^CD25+ regulatory T cells are essential components of the memory-protective immunity to *Candida albicans*. J Immunol. (2002) 169:6298–308. 10.4049/jimmunol.169.11.6298 12444136

[83] Pandiyan P Conti HR Zheng L Peterson AC Mathern DR Hernandez-Santos N. CD4(+)CD25(+)Foxp3(+) regulatory T cells promote Th17 cells in vitro and enhance host resistance in mouse *Candida albicans* Th17 cell infection model. Immunity. (2011) 34:422–34. 10.1016/j.immuni.2011.03.002 21435589 PMC3258585

[84] Montagnoli C Fallarino F Gaziano R Bozza S Bellocchio S Zelante T. Immunity and tolerance to Aspergillus involve functionally distinct regulatory T cells and tryptophan catabolism. J Immunol. (2006) 176:1712–23. 10.4049/jimmunol.176.3.1712 16424201

[85] Hori S Carvalho TL Demengeot J. CD25+CD4^+^ regulatory T cells suppress CD4^+^ T cell-mediated pulmonary hyperinflammation driven by Pneumocystis carinii in immunodeficient mice. Eur J Immunol. (2002) 32:1282–91. 10.1002/1521-4141(200205)32:5<1282::AID-IMMU1282>3.0.CO;2-# 11981815

[86] Scott-Browne JP Shafiani S Tucker-Heard G Ishida-Tsubota K Fontenot JD Rudensky AY. Expansion and function of Foxp3-expressing T regulatory cells during tuberculosis. J Exp Med. (2007) 204:2159–69. 10.1084/jem.20062105 17709423 PMC2118702

[87] Chen CY Huang D Yao S Halliday L Zeng G Wang RC. IL-2 simultaneously expands Foxp3+ T regulatory and T effector cells and confers resistance to severe tuberculosis (TB): implicative Treg-T effector cooperation in immunity to TB. J Immunol. (2012) 188:4278–88. 10.4049/jimmunol.1101291 22474020 PMC3412415

[88] Conti HR Shen F Nayyar N Stocum E Sun JN Lindemann MJ. Th17 cells and IL-17 receptor signaling are essential for mucosal host defense against oral candidiasis. J Exp Med. (2009) 206:299–311. 10.1084/jem.20081463 19204111 PMC2646568

[89] Allam JP Duan Y Winter J Stojanovski G Fronhoffs F Wenghoefer M. Tolerogenic T cells, Th1/Th17 cytokines and TLR2/TLR4 expressing dendritic cells predominate the microenvironment within distinct oral mucosal sites. Allergy. (2011) 66:532–9. 10.1111/j.1398-9995.2010.02510.x 21087216

[90] Cheng SC van de Veerdonk FL Lenardon M Stoffels M Plantinga T Smeekens S. The dectin-1/inflammasome pathway is responsible for the induction of protective T-helper 17 responses that discriminate between yeasts and hyphae of *Candida albicans*. J Leukoc Biol. (2011) 90:357–66. 10.1189/jlb.1210702 21531876 PMC3513931

[91] Bhaskaran N Liu Z Saravanamuthu SS Yan C Hu Y Dong L. Identification of Casz1 as a regulatory protein controlling T helper cell differentiation, inflammation, and immunity. Front Immunol. (2018) 9:184. 10.3389/fimmu.2018.00184 29467767 PMC5808336

[92] Martinez GJ Nurieva RI Yang XO Dong C. Regulation and function of proinflammatory TH17 cells. Ann NY Acad Sci. (2008) 1143:188–211. 10.1196/annals.1443.021 19076351 PMC5793850

[93] Miossec P Kolls JK. Targeting IL-17 and TH17 cells in chronic inflammation. Nat Rev Drug Discov. (2012) 11:763–76. 10.1038/nrd3794 23023676

[94] Pandiyan P Yang XP Saravanamuthu SS Zheng L Ishihara S O'Shea JJ. The role of IL-15 in activating STAT5 and fine-tuning IL-17A production in CD4 T lymphocytes. J Immunol. (2012) 189:4237–46. 10.4049/jimmunol.1201476 22993203 PMC3647038

[95] Duhen R Glatigny S Arbelaez CA Blair TC Oukka M Bettelli E. Cutting edge: the pathogenicity of IFN-gamma-producing Th17 cells is independent of T-bet. J Immunol. (2013) 190:4478–82. 10.4049/jimmunol.1203172 23543757 PMC3633668

[96] Ivanov II Atarashi K Manel N Brodie EL Shima T Karaoz U. Induction of intestinal Th17 cells by segmented filamentous bacteria. Cell. (2009) 139:485–98. 10.1016/j.cell.2009.09.033 19836068 PMC2796826

[97] Littman DR Pamer EG. Role of the commensal microbiota in normal and pathogenic host immune responses. Cell Host Microbe. (2011) 10:311–23. 10.1016/j.chom.2011.10.004 22018232 PMC3202012

[98] Atarashi K Tanoue T Oshima K Suda W Nagano Y Nishikawa H. Treg induction by a rationally selected mixture of Clostridia strains from the human microbiota. Nature. (2013) 500:232–6. 10.1038/nature12331 23842501

[99] Furusawa Y Obata Y Fukuda S Endo TA Nakato G Takahashi D. Commensal microbe-derived butyrate induces the differentiation of colonic regulatory T cells. Nature. (2013) 504:446–50. 10.1038/nature12721 24226770

[100] Singh N Gurav A Sivaprakasam S Brady E Padia R Shi H. Activation of Gpr109a, receptor for niacin and the commensal metabolite butyrate, suppresses colonic inflammation and carcinogenesis. Immunity. (2014) 40:128–39. 10.1016/j.immuni.2013.12.007 24412617 PMC4305274

[101] Bettelli E Korn T Kuchroo VK. Th17: the third member of the effector T cell trilogy. Curr Opin Immunol. (2007) 19:652–7. 10.1016/j.coi.2007.07.020 17766098 PMC2288775

[102] Jiang Y Liu Y Lu H Sun SC Jin W Wang X. Epigenetic activation during T helper 17 cell differentiation is mediated by Tripartite motif containing 28. Nat Commun. (2018) 9:1424. 10.1038/s41467-018-03852-2 29651155 PMC5897371

[103] Ubeda C Taur Y Jenq RR Equinda MJ Son T Samstein M. Vancomycin-resistant Enterococcus domination of intestinal microbiota is enabled by antibiotic treatment in mice and precedes bloodstream invasion in humans. J Clin Invest. (2010) 120:4332–41. 10.1172/JCI43918 21099116 PMC2993598

[104] Elinav E Strowig T Kau AL Henao-Mejia J Thaiss CA Booth CJ. NLRP6 inflammasome regulates colonic microbial ecology and risk for colitis. Cell. (2011) 145:745–57. 10.1016/j.cell.2011.04.022 21565393 PMC3140910

[105] Rehman A Sina C Gavrilova O Hasler R Ott S Baines JF. Nod2 is essential for temporal development of intestinal microbial communities. Gut. (2011) 60:1354–62. 10.1136/gut.2010.216259 21421666

[106] David LA Maurice CF Carmody RN Gootenberg DB Button JE Wolfe BE. Diet rapidly and reproducibly alters the human gut microbiome. Nature. (2014) 505:559–63. 10.1038/nature12820 24336217 PMC3957428

[107] Zhang H Sparks JB Karyala SV Settlage R Luo XM. Host adaptive immunity alters gut microbiota. ISME J. (2015) 9:770–81. 10.1038/ismej.2014.165 25216087 PMC4331585

[108] Bohnhoff MMC (1962). Enhanced susceptibility to Salmonella infection in streptomycin-treated mice. J Infect Dis 111:11. 10.1093/infdis/111.2.117 13968487

[109] Chen X Katchar K Goldsmith J Nanthakumar N Cheknis A Gerding DN. A mouse model of *Clostridium* difficile-associated dieases. Gastroenterology. (2008) 135:1984–92. 10.1053/j.gastro.2008.09.002 18848941

[110] Reeves AE Koenigsknecht MJ Bergin IL Young VB. Suppression of *Clostridium* difficile in the gastrointestinal tracts of germfree mice inoculated with a murine isolate from the family Lachnospiraceae. Infect Immun. (2012) 80:3786–94. 10.1128/IAI.00647-12 22890996 PMC3486043

[111] Sekirov I Tam NM Jogova M Robertson ML Li Y Lupp C. Antibiotic-induced perturbations of the intestinal microbiota alter host susceptibility to enteric infection. Infect Immun. (2008) 76:4726–36. 10.1128/IAI.00319-08 18678663 PMC2546810

[112] Ng KM Ferreyra JA Higginbottom SK Lynch JB Kashyap PC Gopinath S. Microbiota-liberated host sugars facilitate post-antibiotic expansion of enteric pathogens. Nature. (2013) 502:96–9. 10.1038/nature12503 23995682 PMC3825626

[113] Cho H Jaime H de Oliveira RP Kang B Spolski R Vaziri T. Defective IgA response to atypical intestinal commensals in IL-21 receptor deficiency reshapes immune cell homeostasis and mucosal immunity. Mucosal Immunol. (2018). 10.1038/s41385-018-0056-x 30087442 PMC6301133

[114] Bar AK Phukan N Pinheiro J Simoes-Barbosa A. The interplay of host microbiota and parasitic protozoans at mucosal interfaces: implications for the outcomes of infections and diseases. PLoS Negl Trop Dis. (2015) 9:e0004176. 10.1371/journal.pntd.0004176 26658061 PMC4684208

[115] Benson A Pifer R Behrendt CL Hooper LV Yarovinsky F. Gut commensal bacteria direct a protective immune response against *Toxoplasma gondii*. Cell Host Microbe. (2009) 6:187–96. 10.1016/j.chom.2009.06.005 19683684 PMC2746820

[116] Hand TW Dos Santos LM Bouladoux N Molloy MJ Pagan AJ Pepper M. Acute gastrointestinal infection induces long-lived microbiota-specific T cell responses. Science. (2012) 337:1553–6. 10.1126/science.1220961 22923434 PMC3784339

[117] Nilsson J Boasso A Velilla PA Zhang R Vaccari M Franchini G. HIV-1-driven regulatory T-cell accumulation in lymphoid tissues is associated with disease progression in HIV/AIDS. Blood. (2006) 108:3808–17. 10.1182/blood-2006-05-021576 16902147 PMC1895475

[118] Baker CA Clark R Ventura F Jones NG Guzman D Bangsberg DR. Peripheral CD4 loss of regulatory T cells is associated with persistent viraemia in chronic HIV infection. Clin Exp Immunol. (2007) 147:533–9. 10.1111/j.1365-2249.2006.03319.x 17302904 PMC1810503

[119] Kinter AL Hennessey M Bell A Kern S Lin Y Daucher M. CD25(+)CD4(+) regulatory T cells from the peripheral blood of asymptomatic HIV-infected individuals regulate CD4(+) and CD8(+) HIV-specific T cell immune responses *in vitro* and are associated with favorable clinical markers of disease status. J Exp Med. (2004) 200:331–43. 10.1084/jem.20032069 15280419 PMC2211981

[120] Kornfeld C Ploquin MJ Pandrea I Faye A Onanga R Apetrei C. Antiinflammatory profiles during primary SIV infection in African green monkeys are associated with protection against AIDS. J Clin Invest. (2005) 115:1082–91. 10.1172/JCI23006 15761496 PMC1062895

[121] Mozos A Garrido M Carreras J Plana M Diaz A Alos L. Redistribution of FOXP3-positive regulatory T cells from lymphoid tissues to peripheral blood in HIV-infected patients. J Acquir Immune Defic Syndr. (2007) 46:529–37. 10.1097/QAI.0b013e31815b69ae 18193494

[122] Tenorio AR Martinson J Pollard D Baum L Landay A. The relationship of T-regulatory cell subsets to disease stage, immune activation, and pathogen-specific immunity in HIV infection. J Acquir Immune Defic Syndr. (2008) 48:577–80. 10.1097/QAI.0b013e31817bbea5 18645514

[123] Jiao Y Fu J Xing S Fu B Zhang Z Shi M The decrease of regulatory T cells correlates with excessive activation and apoptosis of CD8^+^ T cells in HIV-1-infected typical progressors, but not in long-term non-progressors. Immunology. (2009) 128:e366–375. 10.1111/j.1365-2567.2008.02978.x 19016904 PMC2753909

[124] Owen RE Heitman JW Hirschkorn DF Lanteri MC Biswas HH Martin JN. HIV+ elite controllers have low HIV-specific T-cell activation yet maintain strong, polyfunctional T-cell responses. AIDS. (2010) 24:1095–105. 10.1097/QAD.0b013e3283377a1e 20400885 PMC2972651

[125] Angin M Kwon DS Streeck H Wen F King M Rezai A. Preserved function of regulatory T cells in chronic HIV-1 infection despite decreased numbers in blood and tissue. J Infect Dis. (2012) 205:1495–500. 10.1093/infdis/jis236 22427677 PMC3415814

[126] Favre D Mold J Hunt PW Kanwar B Loke P Seu L. Tryptophan catabolism by indoleamine 2,3-dioxygenase 1 alters the balance of TH17 to regulatory T cells in HIV disease. Sci Transl Med. (2010) 2:32ra36. 10.1126/scitranslmed.3000632 20484731 PMC3034445

[127] Mavigner M Cazabat M Dubois M L'Faqihi F. E. Requena M Pasquier C. (2012). Altered CD4^+^ T cell homing to the gut impairs mucosal immune reconstitution in treated HIV-infected individuals. J Clin Invest. 122, 62–69. 10.1172/JCI59011 22156200 PMC3248296

[128] Brenchley JM. Mucosal immunity in human and simian immunodeficiency lentivirus infections. Mucosal Immunol. (2013) 6:657–65. 10.1038/mi.2013.15 23549448 PMC4154146

[129] Kim CJ McKinnon LR Kovacs C Kandel G Huibner S Chege D. Mucosal Th17 cell function is altered during HIV infection and is an independent predictor of systemic immune activation. J Immunol. (2013) 191:2164–73. 10.4049/jimmunol.1300829 23894197

[130] Pallikkuth S Micci L Ende ZS Iriele RI Cervasi B Lawson B. Maintenance of intestinal Th17 cells and reduced microbial translocation in SIV-infected rhesus macaques treated with interleukin (IL)-21. PLoS Pathog. (2013) 9:e1003471. 10.1371/journal.ppat.1003471 23853592 PMC3701718

[131] Klase Z Ortiz A Deleage C Mudd JC Quinones M Schwartzman E. Dysbiotic bacteria translocate in progressive SIV infection. Mucosal Immunol. (2015) 8:1009–20. 10.1038/mi.2014.128 25586559 PMC4501910

[132] Hensley-McBain T Berard AR Manuzak JA Miller CJ Zevin AS Polacino P. Intestinal damage precedes mucosal immune dysfunction in SIV infection. Mucosal Immunol. (2018) 11:1429–40. 10.1038/s41385-018-0032-5 29907866 PMC6162106

[133] Maloy KJ Powrie F. Intestinal homeostasis and its breakdown in inflammatory bowel disease. Nature. (2011) 474:298–306. 10.1038/nature10208 21677746

[134] Zhang M Sun K Wu Y Yang Y Tso P Wu Z. Interactions between intestinal microbiota and host immune response in inflammatory bowel disease. Front Immunol. (2017) 8:942. 10.3389/fimmu.2017.00942 28855901 PMC5558048

[135] Kabat AM Pott J Maloy KJ. The mucosal immune system and its regulation by autophagy. Front Immunol. (2016) 7:240. 10.3389/fimmu.2016.00240 27446072 PMC4916208

[136] Imhann F Vich Vila A Bonder MJ Fu J Gevers D Visschedijk MC. Interplay of host genetics and gut microbiota underlying the onset and clinical presentation of inflammatory bowel disease. Gut. (2018) 67:108–19. 10.1136/gutjnl-2016-312135 27802154 PMC5699972

[137] Slack E Hapfelmeier S Stecher B Velykoredko Y Stoel M Lawson MA. Innate and adaptive immunity cooperate flexibly to maintain host-microbiota mutualism. Science. (2009) 325:617–20. 10.1126/science.1172747 19644121 PMC3730530

[138] Feng T Cao AT Weaver CT Elson CO Cong Y. Interleukin-12 converts Foxp3+ regulatory T cells to interferon-gamma-producing Foxp3+ T cells that inhibit colitis. Gastroenterology. (2011) 140:2031–43. 10.1053/j.gastro.2011.03.009 21419767 PMC3109200

[139] Rivas MN Koh YT Chen A Nguyen A Lee YH Lawson G. MyD88 is critically involved in immune tolerance breakdown at environmental interfaces of Foxp3-deficient mice. J Clin Invest. (2012) 122:1933–47. 10.1172/JCI40591 22466646 PMC3336968

[140] Iliev ID Funari VA Taylor KD Nguyen Q Reyes CN Strom SP. Interactions between commensal fungi and the C-type lectin receptor Dectin-1 influence colitis. Science. (2012) 336:1314–7. 10.1126/science.1221789 22674328 PMC3432565

[141] Ohnmacht C Park JH Cording S Wing JB Atarashi K Obata Y. MUCOSAL IMMUNOLOGY. The microbiota regulates type 2 immunity through RORgammat+ T cells. Science. (2015) 349:989–93. 10.1126/science.aac4263 26160380

[142] Sefik E Geva-Zatorsky N Oh S Konnikova L Zemmour D McGuire AM. MUCOSAL IMMUNOLOGY. Individual intestinal symbionts induce a distinct population of RORgamma(+) regulatory T cells. Science. (2015) 349:993–7. 10.1126/science.aaa9420 26272906 PMC4700932

[143] Yang BH Hagemann S Mamareli P Lauer U Hoffmann U Beckstette M. Foxp3+ T cells expressing RORgammat represent a stable regulatory T-cell effector lineage with enhanced suppressive capacity during intestinal inflammation. Mucosal Immunol. (2016) 9:444–57. 10.1038/mi.2015.74 26307665

[144] Kim BS Lu H Ichiyama K Chen X Zhang YB Mistry NA. Generation of RORgammat(+) Antigen-Specific T Regulatory 17 Cells from Foxp3(+) Precursors in Autoimmunity. Cell Rep. (2017) 21:195–207. 10.1016/j.celrep.2017.09.021 28978473 PMC5716359

[145] Yang J Zou M Pezoldt J Zhou X Huehn J. Thymus-derived Foxp3(+) regulatory T cells upregulate RORgammat expression under inflammatory conditions. J Mol Med. (2018) 96:1387–94. 10.1007/s00109-018-1706-x 30357435

[146] Mazmanian SK Round JL Kasper DL. A microbial symbiosis factor prevents intestinal inflammatory disease. Nature. (2008) 453:620–5. 10.1038/nature07008 18509436

[147] Arpaia N Campbell C Fan X Dikiy S van der Veeken J deRoos P. Metabolites produced by commensal bacteria promote peripheral regulatory T-cell generation. Nature. (2013) 504:451–5. 10.1038/nature12726 24226773 PMC3869884

[148] Campbell C Dikiy S Bhattarai SK Chinen T Matheis F Calafiore M. Extrathymically generated regulatory T cells establish a niche for intestinal border-dwelling bacteria and affect physiologic metabolite balance. Immunity. (2018) 48:1245–1257 e1249. 10.1016/j.immuni.2018.04.013 29858010 PMC6260932

[149] Tang C Kakuta S Shimizu K Kadoki M Kamiya T Shimazu T Suppression of IL-17F, but not of IL-17A, provides protection against colitis by inducing Treg cells through modification of the intestinal microbiota. Nat Immunol. (2018) 19:755–65. 10.1038/s41590-018-0134-y 29915298

[150] Sultan AS Kong EF Rizk AM Jabra-Rizk MA. The oral microbiome: a Lesson in coexistence. PLoS Pathog. (2018) 14:e1006719. 10.1371/journal.ppat.1006719 29370304 PMC5784999

[151] Aas JA Paster BJ Stokes LN Olsen I Dewhirst FE. Defining the normal bacterial flora of the oral cavity. J Clin Microbiol. (2005) 43:5721–32. 10.1128/JCM.43.11.5721-5732.2005 16272510 PMC1287824

[152] Avila M Ojcius DM Yilmaz O. The oral microbiota: living with a permanent guest. DNA Cell Biol. (2009) 28:405–11. 10.1089/dna.2009.0874 19485767 PMC2768665

[153] Zaura E Keijser BJ Huse SM Crielaard W. Defining the healthy “core microbiome” of oral microbial communities. BMC Microbiol. (2009) 9:259. 10.1186/1471-2180-9-259 20003481 PMC2805672

[154] Ghannoum MA Jurevic RJ Mukherjee PK Cui F Sikaroodi M Naqvi A. Characterization of the oral fungal microbiome (mycobiome) in healthy individuals. PLoS Pathog. (2010) 6:e1000713. 10.1371/journal.ppat.1000713 20072605 PMC2795202

[155] Ahn J Yang L Paster BJ Ganly I Morris L Pei Z. Oral microbiome profiles: 16S rRNA pyrosequencing and microarray assay comparison. PLoS ONE. (2011) 6:e22788. 10.1371/journal.pone.0022788 21829515 PMC3146496

[156] Belda-Ferre P Alcaraz LD Cabrera-Rubio R Romero H Simon-Soro A Pignatelli M. The oral metagenome in health and disease. ISME J. (2012) 6:46–56. 10.1038/ismej.2011.85 21716308 PMC3246241

[157] Dang AT Cotton S Sankaran-Walters S Li CS Lee CY Dandekar S. Evidence of an increased pathogenic footprint in the lingual microbiome of untreated HIV infected patients. BMC Microbiol. (2012) 12:153. 10.1186/1471-2180-12-153 22838383 PMC3438044

[158] Yamazaki S Maruyama A Okada K Matsumoto M Morita A Seya T. Dendritic cells from oral cavity induce Foxp3(+) regulatory T cells upon antigen stimulation. PLoS ONE. (2012) 7:e51665. 10.1371/journal.pone.0051665 23272135 PMC3525649

[159] Bashan A Gibson TE Friedman J Carey VJ Weiss ST Hohmann EL. Universality of human microbial dynamics. Nature. (2016) 534:259–62. 10.1038/nature18301 27279224 PMC4902290

[160] Teng F Darveekaran Nair SS Zhu P Li S Huang S Li X. Impact of DNA extraction method and targeted 16S-rRNA hypervariable region on oral microbiota profiling. Sci Rep. (2018) 8:16321. 10.1038/s41598-018-34294-x 30397210 PMC6218491

[161] Shirtliff ME Peters BM Jabra-Rizk MA. Cross-kingdom interactions: *Candida albicans* and bacteria. FEMS Microbiol Lett. (2009) 299:1–8. 10.1111/j.1574-6968.2009.01668.x 19552706 PMC4406406

[162] Peters BM Jabra-Rizk MA O'May GA Costerton JW Shirtliff ME. Polymicrobial interactions: impact on pathogenesis and human disease. Clin Microbiol Rev. (2012) 25:193–213. 10.1128/CMR.00013-11 22232376 PMC3255964

[163] Wright CJ Burns LH Jack AA Back CR Dutton LC Nobbs AH. Microbial interactions in building of communities. Mol Oral Microbiol. (2013) 28:83–101. 10.1111/omi.12012 23253299 PMC3600090

[164] Guo L He X Shi W. Intercellular communications in multispecies oral microbial communities. Front Microbiol. (2014) 5:328. 10.3389/fmicb.2014.00328 25071741 PMC4076886

[165] Murray JL Connell JL Stacy A Turner KH Whiteley M. Mechanisms of synergy in polymicrobial infections. J Microbiol. (2014) 52:188–99. 10.1007/s12275-014-4067-3 24585050 PMC7090983

[166] Xu H Sobue T Thompson A Xie Z Poon K Ricker A. Streptococcal co-infection augments *Candida* pathogenicity by amplifying the mucosal inflammatory response. Cell Microbiol. (2014) 16:214–31. 10.1111/cmi.12216 24079976 PMC3956708

[167] Cutler CW Jotwani R. Dendritic cells at the oral mucosal interface. J Dent Res. (2006) 85:678–89. 10.1177/154405910608500801 16861283 PMC2254185

[168] Novak N Haberstok J Bieber T Allam JP. The immune privilege of the oral mucosa. Trends Mol Med. (2008) 14:191–8. 10.1016/j.molmed.2008.03.001 18396104

[169] Hovav AH. Dendritic cells of the oral mucosa. Mucosal Immunol. (2013) 7:27–37. 10.1038/mi.2013.42 23757304

[170] Pandiyan P Bhaskaran N Zhang Y Weinberg A. Isolation of T cells from mouse oral tissues. Biol Proced Online. (2014) 16:4. 10.1186/1480-9222-16-4 24612879 PMC3984730

[171] Park JY Chung H DiPalma DT Tai X Park JH. Immune quiescence in the oral mucosa is maintained by a uniquely large population of highly activated Foxp3(+) regulatory T cells. Mucosal Immunol. (2018). 10.1038/s41385-018-0027-2 29743613 PMC6035783

[172] Milner JD Brenchley JM Laurence A Freeman AF Hill BJ Elias KM. Impaired T(H)17 cell differentiation in subjects with autosomal dominant hyper-IgE syndrome. Nature. (2008) 452:773–6. 10.1038/nature06764 18337720 PMC2864108

[173] Blaschitz C Raffatellu M. Th17 cytokines and the gut mucosal barrier. J Clin Immunol. (2010) 30:196–203. 10.1007/s10875-010-9368-7 20127275 PMC2842875

[174] Darveau RP. Periodontitis: a polymicrobial disruption of host homeostasis. Nat Rev Microbiol. (2010) 8:481–90. 10.1038/nrmicro2337 20514045

[175] Pion M Jaramillo-Ruiz D Martinez A Munoz-Fernandez MA Correa-Rocha R. HIV infection of human regulatory T cells downregulates Foxp3 expression by increasing DNMT3b levels and DNA methylation in the FOXP3 gene. AIDS. (2013) 27:2019–29. 10.1097/QAD.0b013e32836253fd 24201117

[176] Cheng WC Hughes FJ Taams LS. The presence, function and regulation of IL-17 and Th17 cells in periodontitis. J Clin Periodontol. (2014) 41:541–9. 10.1111/jcpe.12238 24735470

[177] Bhaskaran N Quigley C Weinberg A Huang A Popkin D Pandiyan P. Transforming growth factor-beta1 sustains the survival of Foxp3 regulatory cells during late phase of oropharyngeal candidiasis infection. Mucosal Immunol. (2016) 9:1015–26. 10.1038/mi.2015.115 26530137 PMC4854793

[178] Bhaskaran N Weinberg A Pandiyan P. Th17 inflammation model of oropharyngeal candidiasis in immunodeficient mice. J Vis Exp. (2015). 10.3791/52538 25742163 PMC4354660

[179] Hernandez-Santos N Huppler AR Peterson AC Khader SA McKenna KC Gaffen SL. Th17 cells confer long-term adaptive immunity to oral mucosal *Candida albicans* infections. Mucosal Immunol. (2013) 6:900–10. 10.1038/mi.2012.128 23250275 PMC3608691

[180] Whibley N Gaffen SL. Brothers in arms: Th17 and Treg responses in *Candida albicans* immunity. PLoS Pathog. (2014) 10:e1004456. 10.1371/journal.ppat.1004456 25474407 PMC4256420

[181] Khan SA Kong EF Meiller TF Jabra-Rizk MA. Periodontal diseases: bug induced, host promoted. PLoS Pathog. (2015) 11:e1004952. 10.1371/journal.ppat.1004952 26226364 PMC4520614

[182] Mahanonda R Champaiboon C Subbalekha K Sa-Ard-Iam N Yongyuth A Isaraphithakkul B. Memory T cell subsets in healthy gingiva and periodontitis tissues. J Periodontol. (2018) 89:1121–30. 10.1002/JPER.17-0674 29790576

[183] Tamai R Sugamata M Kiyoura Y. *Candida albicans* enhances invasion of human gingival epithelial cells and gingival fibroblasts by *Porphyromonas gingivalis*. Microb Pathog. (2011) 51:250–4. 10.1016/j.micpath.2011.06.009 21742026

[184] Canabarro A Valle C Farias MR Santos FB Lazera M Wanke B. Association of subgingival colonization of *Candida albicans* and other yeasts with severity of chronic periodontitis. J Periodontal Res. (2013) 48:428–32. 10.1111/jre.12022 23137301

[185] Okui T Aoki Y Ito H Honda T Yamazaki K. The presence of IL-17+/FOXP3+ double-positive cells in periodontitis. J Dent Res. (2012) 91:574–9. 10.1177/0022034512446341 22522772

[186] Andersson J Fehniger TE Patterson BK Pottage J Agnoli M Jones P. Early reduction of immune activation in lymphoid tissue following highly active HIV therapy. AIDS. (1998) 12:F123–129. 9708402 10.1097/00002030-199811000-00004

[187] Starr JR Huang Y Lee KH Murphy CM Moscicki AB Shiboski CH. Oral microbiota in youth with perinatally acquired HIV infection. Microbiome. (2018) 6:100. 10.1186/s40168-018-0484-6 29855347 PMC5984365

[188] Bassis CM Erb-Downward JR Dickson RP Freeman CM Schmidt TM Young VB. Analysis of the upper respiratory tract microbiotas as the source of the lung and gastric microbiotas in healthy individuals. MBio. (2015) 6:e00037. 10.1128/mBio.00037-15 25736890 PMC4358017

[189] Dickson RP Erb-Downward JR Freeman CM McCloskey L Beck JM Huffnagle GB. Spatial variation in the healthy human lung microbiome and the adapted island model of lung biogeography. Ann Am Thorac Soc. (2015) 12:821–30. 10.1513/AnnalsATS.201501-029OC 25803243 PMC4590020

[190] Teo SM Mok D Pham K Kusel M Serralha M Troy N. The infant nasopharyngeal microbiome impacts severity of lower respiratory infection and risk of asthma development. Cell Host Microbe. (2015) 17:704–15. 10.1016/j.chom.2015.03.008 25865368 PMC4433433

[191] Hilty M Burke C Pedro H Cardenas P Bush A Bossley C. Disordered microbial communities in asthmatic airways. PLoS ONE. (2010) 5:e8578. 10.1371/journal.pone.0008578 20052417 PMC2798952

[192] Huang YJ Nariya S Harris JM Lynch SV Choy DF Arron JR. The airway microbiome in patients with severe asthma: associations with disease features and severity. J Allergy Clin Immunol. (2015) 136:874–84. 10.1016/j.jaci.2015.05.044 26220531 PMC4600429

[193] Madan JC Koestler DC Stanton BA Davidson L Moulton LA Housman ML. Serial analysis of the gut and respiratory microbiome in cystic fibrosis in infancy: interaction between intestinal and respiratory tracts and impact of nutritional exposures. MBio. (2012) 3:e00251–12. 10.1128/mBio.00251-12 22911969 PMC3428694

[194] Krone CL Biesbroek G Trzcinski K Sanders EA Bogaert D. Respiratory microbiota dynamics following *Streptococcus pneumoniae* acquisition in young and elderly mice. Infect Immun. (2014) 82:1725–31. 10.1128/IAI.01290-13 24516113 PMC3993406

[195] Ma B Forney LJ Ravel J. Vaginal microbiome: rethinking health and disease. Annu Rev Microbiol. (2012) 66:371–89. 10.1146/annurev-micro-092611-150157 22746335 PMC3780402

[196] Nemati M Malla N Yadav M Khorramdelazad H Jafarzadeh A. Humoral and T cell-mediated immune response against trichomoniasis. Parasite Immunol. (2018) 40:e12510. 10.1111/pim.12510 29266263

[197] Lund JM Hsing L Pham TT Rudensky AY. Coordination of early protective immunity to viral infection by regulatory T cells. Science. (2008) 320:1220–4. 10.1126/science.1155209 18436744 PMC2519146

[198] Soerens AG Da Costa A Lund JM. Regulatory T cells are essential to promote proper CD4 T-cell priming upon mucosal infection. Mucosal Immunol. (2016) 9:1395–406. 10.1038/mi.2016.19 27007674 PMC5035160

[199] St Leger AJ Desai JV Drummond RA Kugadas A Almaghrabi F Silver P. An Ocular Commensal Protects against Corneal Infection by Driving an Interleukin-17 Response from Mucosal gammadelta T Cells. Immunity. (2017) 47:148–58 e145. 10.1016/j.immuni.2017.06.014 28709803 PMC5553552

[200] Mahdavinia M Keshavarzian A Tobin MC Landay AL Schleimer RP. A comprehensive review of the nasal microbiome in chronic rhinosinusitis (CRS). Clin Exp Aller. (2016) 46:21–41. 10.1111/cea.12666 26510171 PMC4715613

[201] Koskinen K Reichert JL Hoier S Schachenreiter J Duller S Moissl-Eichinger C. The nasal microbiome mirrors and potentially shapes olfactory function. Sci Rep. (2018) 8:1296. 10.1038/s41598-018-19438-3 29358754 PMC5778015

[202] Yang HJ LoSavio PS Engen PA Naqib A Mehta A Kota R. Association of nasal microbiome and asthma control in patients with chronic rhinosinusitis. Clin Exp Aller. (2018) 48:1744–7. 10.1111/cea.13255 30126004 PMC6265059

[203] Ritchie ND Ijaz UZ Evans TJ. IL-17 signalling restructures the nasal microbiome and drives dynamic changes following Streptococcus pneumoniae colonization. BMC Genomics. (2017) 18:807. 10.1186/s12864-017-4215-3 29058583 PMC5651609

[204] Oliveira MR Tafuri WL Afonso LC Oliveira MA Nicoli JR Vieira EC. Germ-free mice produce high levels of interferon-gamma in response to infection with Leishmania major but fail to heal lesions. Parasitology. (2005) 131(Pt 4), 477–488. 10.1017/S0031182005008073 16174412

[205] Ley RE Peterson DA Gordon JI. Ecological and evolutionary forces shaping microbial diversity in the human intestine. Cell. (2006) 124:837–48. 10.1016/j.cell.2006.02.017 16497592

[206] Kelly D King T Aminov R. Importance of microbial colonization of the gut in early life to the development of immunity. Mutat Res. (2007) 622:58–69. 10.1016/j.mrfmmm.2007.03.011 17612575

[207] Olszak T An D Zeissig S Vera MP Richter J Franke A. Microbial exposure during early life has persistent effects on natural killer T cell function. Science. (2012) 336:489–93. 10.1126/science.1219328 22442383 PMC3437652

[208] Caballero S Pamer EG. Microbiota-mediated inflammation and antimicrobial defense in the intestine. Annu Rev Immunol. (2015) 33:227–56. 10.1146/annurev-immunol-032713-120238 25581310 PMC4540477

[209] Thiemann S Smit N Strowig T. Antibiotics and the intestinal microbiome: individual responses, resilience of the ecosystem, and the susceptibility to infections. Curr Topics Microbiol Immunol. (2016) 398:124–38. 10.1007/82_2016_504 27738912

[210] Sorg JA Sonenshein AL. Bile salts and glycine as cogerminants for *Clostridium* difficile spores. J Bacteriol. (2008) 190:2505–12. 10.1128/JB.01765-07 18245298 PMC2293200

[211] Vaishnava S Behrendt CL Ismail AS Eckmann L Hooper LV. Paneth cells directly sense gut commensals and maintain homeostasis at the intestinal host-microbial interface. Proc Natl Acad Sci USA. (2008) 105:20858–63. 10.1073/pnas.0808723105 19075245 PMC2603261

[212] Sun Y O'Riordan MX. Regulation of bacterial pathogenesis by intestinal short-chain Fatty acids. Adv Appl Microbiol. (2013) 85:93–118. 10.1016/B978-0-12-407672-3.00003-4 23942149 PMC4029053

[213] Thiemann S Smit N Roy U Lesker TR Galvez EJC Helmecke J. Enhancement of IFNgamma production by distinct commensals ameliorates salmonella-induced disease. Cell Host Microbe. (2017) 21:682–94 e685. 10.1016/j.chom.2017.05.005 28618267

[214] Shukla SD Budden KF Neal R Hansbro PM. Microbiome effects on immunity, health and disease in the lung. Clin Transl Immunol. (2017) 6:e133. 10.1038/cti.2017.6 28435675 PMC5382435

[215] Round JL Lee SM Li J Tran G Jabri B Chatila TA. The Toll-like receptor 2 pathway establishes colonization by a commensal of the human microbiota. Science. (2011) 332:974–7. 10.1126/science.1206095 21512004 PMC3164325

[216] Kabat AM Harrison OJ Riffelmacher T Moghaddam AE Pearson CF Laing A. The autophagy gene Atg16l1 differentially regulates Treg and TH2 cells to control intestinal inflammation. Elife. (2016) 5:e12444. 10.7554/eLife.12444 26910010 PMC4798959

[217] Wang S Charbonnier LM Noval Rivas M Georgiev P Li N Gerber G. MyD88 Adaptor-dependent microbial sensing by regulatory t cells promotes mucosal tolerance and enforces commensalism. Immunity. (2015) 43:289–303. 10.1016/j.immuni.2015.06.014 26231118 PMC4545404

[218] Schenten D Nish SA Yu S Yan X Lee HK Brodsky I. Signaling through the adaptor molecule MyD88 in CD4^+^ T cells is required to overcome suppression by regulatory T cells. Immunity. (2014) 40:78–90. 10.1016/j.immuni.2013.10.023 24439266 PMC4445716

[219] Cording S Fleissner D Heimesaat MM Bereswill S Loddenkemper C Uematsu S. Commensal microbiota drive proliferation of conventional and Foxp3(+) regulatory CD4(+) T cells in mesenteric lymph nodes and Peyer's patches. Eur J Microbiol Immunol. (2013) 3:1–10. 10.1556/EuJMI.3.2013.1.1 24265914 PMC3832078

[220] Moor K Diard M Sellin ME Felmy B Wotzka SY Toska A. High-avidity IgA protects the intestine by enchaining growing bacteria. Nature. (2017) 544:498–502. 10.1038/nature22058 28405025

[221] Wu W Sun M Chen F Cao AT Liu H Zhao Y. Microbiota metabolite short-chain fatty acid acetate promotes intestinal IgA response to microbiota which is mediated by GPR43. Mucosal Immunol. (2017) 10:946–56. 10.1038/mi.2016.114 27966553 PMC5471141

[222] Tsuji M Komatsu N Kawamoto S Suzuki K Kanagawa O Honjo T. Preferential generation of follicular B helper T cells from Foxp3+ T cells in gut Peyer's patches. Science. (2009) 323:1488–92. 10.1126/science.1169152 19286559

[223] Kawamoto S Maruya M Kato LM Suda W Atarashi K Doi Y. Foxp3(+) T cells regulate immunoglobulin a selection and facilitate diversification of bacterial species responsible for immune homeostasis. Immunity. (2014) 41:152–65. 10.1016/j.immuni.2014.05.016 25017466

[224] Cao AT Yao S Gong B Elson CO Cong Y. Th17 cells upregulate polymeric Ig receptor and intestinal IgA and contribute to intestinal homeostasis. J Immunol. (2012) 189:4666–73. 10.4049/jimmunol.1200955 22993206 PMC3478497

[225] Hirota K Turner JE Villa M Duarte JH Demengeot J Steinmetz OM. Plasticity of Th17 cells in Peyer's patches is responsible for the induction of T cell-dependent IgA responses. Nat Immunol. (2013) 14:372–9. 10.1038/ni.2552 23475182 PMC3672955

[226] Goto Y Obata T Kunisawa J Sato S Ivanov II Lamichhane A. Innate lymphoid cells regulate intestinal epithelial cell glycosylation. Science. (2014) 345:1254009. 10.1126/science.1254009 25214634 PMC4774895

[227] Goto Y Uematsu S Kiyono H. Epithelial glycosylation in gut homeostasis and inflammation. Nat Immunol. (2016) 17:1244–51. 10.1038/ni.3587 27760104

[228] Hepworth MR Monticelli LA Fung TC Ziegler CG Grunberg S Sinha R. Innate lymphoid cells regulate CD4^+^ T-cell responses to intestinal commensal bacteria. Nature. (2013) 498:113–7. 10.1038/nature12240 23698371 PMC3699860

[229] Victoriano AF Imai K Okamoto T. Interaction between endogenous bacterial flora and latent HIV infection. Clin Vaccine Immunol. (2013) 20:773–9. 10.1128/CVI.00766-12 23616411 PMC3675960

[230] Yu X Shahir AM Sha J Feng Z Eapen B Nithianantham S. Short-chain fatty acids from periodontal pathogens suppress histone deacetylases, EZH2, and SUV39H1 to promote Kaposi's sarcoma-associated herpesvirus replication. J Virol. (2014) 88:4466–79. 10.1128/JVI.03326-13 24501407 PMC3993761

[231] Macfarlane S Macfarlane GT. Regulation of short-chain fatty acid production. Proc Nutr Soc. (2003) 62:67–72. 10.1079/PNS2002207 12740060

[232] Zeng H Chi H. Metabolic control of regulatory T cell development and function. Trends Immunol. (2015) 36:3–12. 10.1016/j.it.2014.08.003 25248463 PMC4280284

[233] Coombes JL Siddiqui KR Arancibia-Carcamo CV Hall J Sun CM Belkaid Y. A functionally specialized population of mucosal CD103+ DCs induces Foxp3+ regulatory T cells via a TGF-beta and retinoic acid-dependent mechanism. J Exp Med. (2007) 204:1757–64. 10.1084/jem.20070590 17620361 PMC2118683

[234] Iliev ID Mileti E Matteoli G Chieppa M Rescigno M. Intestinal epithelial cells promote colitis-protective regulatory T-cell differentiation through dendritic cell conditioning. Mucosal Immunol. (2009) 2:340–50. 10.1038/mi.2009.13 19387433

[235] Denning TL Wang YC Patel SR Williams IR Pulendran B. Lamina propria macrophages and dendritic cells differentially induce regulatory and interleukin 17-producing T cell responses. Nat Immunol. (2007) 8:1086–94. 10.1038/ni1511 17873879

[236] Molenaar R Knippenberg M Goverse G Olivier BJ de Vos AF O'Toole T. Expression of retinaldehyde dehydrogenase enzymes in mucosal dendritic cells and gut-draining lymph node stromal cells is controlled by dietary vitamin A. J Immunol. (2011) 186:1934–42. 10.4049/jimmunol.1001672 21220692

[237] Goverse G Molenaar R Macia L Tan J Erkelens MN Konijn T Diet-derived short chain fatty acids stimulate intestinal epithelial cells to induce mucosal tolerogenic dendritic cells. J Immunol. (2017) 198:2172–81. 10.4049/jimmunol.1600165 28100682

[238] Atarashi K Suda W Luo C Kawaguchi T Motoo I Narushima S. Ectopic colonization of oral bacteria in the intestine drives TH1 cell induction and inflammation. Science. (2017) 358:359–65. 10.1126/science.aan4526 29051379 PMC5682622

[239] Noverr MC Noggle RM Toews GB Huffnagle GB. Role of antibiotics and fungal microbiota in driving pulmonary allergic responses. Infect Immun. (2004) 72:4996–5003. 10.1128/IAI.72.9.4996-5003.2004 15321991 PMC517468

[240] Samuelson DR Welsh DA Shellito JE. Regulation of lung immunity and host defense by the intestinal microbiota. Front Microbiol. (2015) 6:1085. 10.3389/fmicb.2015.01085 26500629 PMC4595839

[241] Park J Kim M Kang SG Jannasch AH Cooper B Patterson J. Short-chain fatty acids induce both effector and regulatory T cells by suppression of histone deacetylases and regulation of the mTOR-S6K pathway. Mucosal Immunol. (2015) 8:80–93. 10.1038/mi.2014.44 24917457 PMC4263689

[242] Datta SR Brunet A Greenberg ME. Cellular survival: a play in three Akts. Genes Dev. (1999) 13:2905–27. 10579998 10.1101/gad.13.22.2905

[243] Pandiyan P Gartner D Soezeri O Radbruch A Schulze-Osthoff K Brunner-Weinzierl MC. CD152 (CTLA-4) determines the unequal resistance of Th1 and Th2 cells against activation-induced cell death by a mechanism requiring PI3 kinase function. J Exp Med. (2004) 199:831–42. 10.1084/jem.20031058 15007096 PMC2212725

[244] Delgoffe GM Kole TP Zheng Y Zarek PE Matthews KL Xiao B. The mTOR kinase differentially regulates effector and regulatory T cell lineage commitment. Immunity. (2009) 30:832–44. 10.1016/j.immuni.2009.04.014 19538929 PMC2768135

[245] Esposito M Ruffini F Bellone M Gagliani N Battaglia M Martino G. Rapamycin inhibits relapsing experimental autoimmune encephalomyelitis by both effector and regulatory T cells modulation. J Neuroimmunol. (2010) 220:52–63. 10.1016/j.jneuroim.2010.01.001 20149931

[246] Procaccini C De Rosa V Galgani M Abanni L Cali G Porcellini A. An oscillatory switch in mTOR kinase activity sets regulatory T cell responsiveness. Immunity. (2010) 33:929–41. 10.1016/j.immuni.2010.11.024 21145759 PMC3133602

[247] Delgoffe GM Pollizzi KN Waickman AT Heikamp E Meyers DJ Horton MR. The kinase mTOR regulates the differentiation of helper T cells through the selective activation of signaling by mTORC1 and mTORC2. Nat Immunol. (2011) 12:295–303. 10.1038/ni.2005 21358638 PMC3077821

[248] So T Croft M. Regulation of PI-3-Kinase and Akt Signaling in T lymphocytes and other cells by TNFR family molecules. Front Immunol. (2013) 4:139. 10.3389/fimmu.2013.00139 23760533 PMC3675380

[249] Kaneda MM Messer KS Ralainirina N Li H Leem CJ Gorjestani S. PI3Kgamma is a molecular switch that controls immune suppression. Nature. (2016) 539:437–42. 10.1038/nature19834 27642729 PMC5479689

[250] Chi H. Regulation and function of mTOR signalling in T cell fate decisions. Nat Rev Immunol. (2012) 12:325–38. 10.1038/nri3198 22517423 PMC3417069

[251] Powell JD Pollizzi KN Heikamp EB Horton MR. Regulation of immune responses by mTOR. Annu Rev Immunol. (2012) 30:39–68. 10.1146/annurev-immunol-020711-075024 22136167 PMC3616892

[252] Sauer S Bruno L Hertweck A Finlay D Leleu M Spivakov M. T cell receptor signaling controls Foxp3 expression via PI3K, Akt, and mTOR. Proc Natl Acad Sci USA. (2008) 105:7797–802. 10.1073/pnas.0800928105 18509048 PMC2409380

[253] Clambey ET McNamee EN Westrich JA Glover LE Campbell EL Jedlicka P. Hypoxia-inducible factor-1 alpha-dependent induction of FoxP3 drives regulatory T-cell abundance and function during inflammatory hypoxia of the mucosa. Proc Natl Acad Sci USA. (2012) 109:E2784–2793. 10.1073/pnas.1202366109 22988108 PMC3478644

[254] Zeng H Yang K Cloer C Neale G Vogel P Chi H. mTORC1 couples immune signals and metabolic programming to establish T(reg)-cell function. Nature. (2013) 499:485–90. 10.1038/nature12297 23812589 PMC3759242

[255] Carbone F De Rosa V Carrieri PB Montella S Bruzzese D Porcellini A. Regulatory T cell proliferative potential is impaired in human autoimmune disease. Nat Med. (2014) 20:69–74. 10.1038/nm.3411 24317118

[256] Hurez V Dao V Liu A Pandeswara S Gelfond J Sun L. Chronic mTOR inhibition in mice with rapamycin alters T, B, myeloid, and innate lymphoid cells and gut flora and prolongs life of immune-deficient mice. Aging Cell. (2015) 14:945–56. 10.1111/acel.12380 26315673 PMC4693453

[257] Shrestha S Yang K Guy C Vogel P Neale G Chi H. Treg cells require the phosphatase PTEN to restrain TH1 and TFH cell responses. Nat Immunol. (2015) 16:178–87. 10.1038/ni.3076 25559258 PMC4297581

[258] Apostolidis SA Rodriguez-Rodriguez N Suarez-Fueyo A Dioufa N Ozcan E Crispin JC. Phosphatase PP2A is requisite for the function of regulatory T cells. Nat Immunol. (2016) 17:556–64. 10.1038/ni.3390 26974206 PMC4837024

[259] Gabriel SS Kallies A. Sugars and fat - A healthy way to generate functional regulatory T cells. Eur J Immunol. (2016) 46:2705–9. 10.1002/eji.201646663 27918097

[260] Kasper IR Apostolidis SA Sharabi A Tsokos GC. Empowering Regulatory T Cells in Autoimmunity. Trends Mol Med. (2016) 22:784–97. 10.1016/j.molmed.2016.07.003 27461103 PMC5003773

[261] Merchant HA Liu F Orlu Gul M Basit AW. Age-mediated changes in the gastrointestinal tract. Int J Pharm. (2016) 512:382–95. 10.1016/j.ijpharm.2016.04.024 27085646

[262] Shibagaki N Suda W Clavaud C Bastien P Takayasu L Iioka E. Aging-related changes in the diversity of women's skin microbiomes associated with oral bacteria. Sci Rep. (2017) 7:10567. 10.1038/s41598-017-10834-9 28874721 PMC5585242

[263] Zhang L Wang Y Xiayu X Shi C Chen W Song N. Altered Gut Microbiota in a Mouse Model of Alzheimer's Disease. J Alzheimers Dis. (2017) 60:1241–57. 10.3233/JAD-170020 29036812

[264] Zhang T Xiang J Cui B He Z Li P Chen H. Cost-effectiveness analysis of fecal microbiota transplantation for inflammatory bowel disease. Oncotarget. (2017) 8:88894–903. 10.18632/oncotarget.21491 29179485 PMC5687655

[265] Zhang W Zhu YH Zhou D Wu Q Song D Dicksved J. Oral administration of a select mixture of bacillus probiotics affects the gut microbiota and goblet cell function following *Escherichia coli* challenge in newly weaned pigs of genotype MUC4 that are supposed to be enterotoxigenic *E. coli* F4ab/ac receptor negative. Appl Environ Microbiol. (2017) 83: e02747–16. 10.1128/AEM.02747-16 27881419 PMC5244294

[266] Bodogai M O'Connell J Kim K Kim Y Moritoh K Chen C. Commensal bacteria contribute to insulin resistance in aging by activating innate B1a cells. Sci Transl Med. (2018) 10:eaat4271. 10.1126/scitranslmed.aat4271 30429354 PMC6445267

[267] Irie K Novince CM Darveau RP. Impact of the oral commensal flora on alveolar bone homeostasis. J Dent Res. (2014) 93:801–6. 10.1177/0022034514540173 24935067 PMC4126224

[268] Irie K Tomofuji T Ekuni D Fukuhara D Uchida Y Kataoka K. Age-related changes of CD4(+) T cell migration and cytokine expression in germ-free and SPF mice periodontium. Arch Oral Biol. (2018) 87:72–8. 10.1016/j.archoralbio.2017.12.007 29274620

[269] Lee YK Menezes JS Umesaki Y Mazmanian SK. Proinflammatory T-cell responses to gut microbiota promote experimental autoimmune encephalomyelitis. Proc Natl Acad Sci USA. (2011) 108(Suppl. 1):4615–22. 10.1073/pnas.1000082107 20660719 PMC3063590

[270] Wu HJ Ivanov II Darce J Hattori K Shima T Umesaki Y. Gut-residing segmented filamentous bacteria drive autoimmune arthritis via T helper 17 cells. Immunity. (2010) 32:815–27. 10.1016/j.immuni.2010.06.001 20620945 PMC2904693

[271] Rogier R Evans-Marin H Manasson J van der Kraan PM Walgreen B Helsen MM. Alteration of the intestinal microbiome characterizes preclinical inflammatory arthritis in mice and its modulation attenuates established arthritis. Sci Rep. (2017) 7:15613. 10.1038/s41598-017-15802-x 29142301 PMC5688157

[272] Rubinstein MR Wang X Liu W Hao Y Cai G Han YW. Fusobacterium nucleatum promotes colorectal carcinogenesis by modulating E-cadherin/beta-catenin signaling via its FadA adhesin. Cell Host Microbe. (2013) 14:195–206. 10.1016/j.chom.2013.07.012 23954158 PMC3770529

[273] Mima K Sukawa Y Nishihara R Qian ZR Yamauchi M Inamura K. *Fusobacterium nucleatum* and T Cells in Colorectal Carcinoma. JAMA Oncol. (2015) 1:653–61. 10.1001/jamaoncol.2015.1377 26181352 PMC4537376

[274] Hamada T Zhang X Mima K Bullman S Sukawa Y Nowak JA. *Fusobacterium nucleatum* in colorectal cancer relates to immune response differentially by tumor microsatellite instability status. Cancer Immunol Res. (2018) 6:1327–36. 10.1158/2326-6066.CIR-18-0174 30228205 PMC6215508

[275] Liu L Tabung FK Zhang X Nowak JA Qian ZR Hamada T. Diets that promote colon inflammation associate with risk of colorectal carcinomas that contain *Fusobacterium nucleatum*. Clin Gastroenterol Hepatol. (2018) 16:1622–1631 e1623. 10.1016/j.cgh.2018.04.030 29702299 PMC6151288

[276] Yang CY Yeh YM Yu HY Chin CY Hsu CW Liu H. Oral microbiota community dynamics associated with oral squamous cell carcinoma staging. Front Microbiol. (2018) 9:862. 10.3389/fmicb.2018.00862 29774014 PMC5943489

[277] Brennan CA Garrett WS. *Fusobacterium nucleatum* - symbiont, opportunist and oncobacterium. Nat Rev Microbiol. (2019) 17:156–166. 10.1038/s41579-018-0129-6 30546113 PMC6589823

[278] Dinakaran V Mandape SN Shuba K Pratap S Sakhare SS Tabatabai MA. Identification of specific oral and gut pathogens in full thickness colon of colitis patients: implications for colon motility. Front Microbiol. (2018) 9:3220. 10.3389/fmicb.2018.03220 30666239 PMC6330997

[279] Sears CL. The who, where and how of fusobacteria and colon cancer. Elife. (2018) 7:e28434. 10.7554/eLife.28434 29533185 PMC5849411

[280] Lee SM Donaldson GP Mikulski Z Boyajian S Ley K Mazmanian SK. Bacterial colonization factors control specificity and stability of the gut microbiota. Nature. (2013) 501:426–9. 10.1038/nature12447 23955152 PMC3893107

[281] D'Souza B Slack T Wong SW Lam F Muhlmann M Koestenbauer J. Randomized controlled trial of probiotics after colonoscopy. ANZ J Surg. (2017) 87:E65–E69. 10.1111/ans.13225 26183594

[282] Bagga D Reichert JL Koschutnig K Aigner CS Holzer P Koskinen K. Probiotics drive gut microbiome triggering emotional brain signatures. Gut Microbes. (2018) 9:486–96. 10.1080/19490976.2018.1460015 29723105 PMC6287679

[283] Shepherd ES DeLoache WC Pruss KM Whitaker WR Sonnenburg JL. An exclusive metabolic niche enables strain engraftment in the gut microbiota. Nature. (2018) 557:434–8. 10.1038/s41586-018-0092-4 29743671 PMC6126907

[284] Hoffmann D Palumbo F Ravel J Roghmann MC Rowthorn V von Rosenvinge E. Improving regulation of microbiota transplants. Science. (2017) 358:1390–1. 10.1126/science.aaq0034 29242336 PMC5773100

[285] Sun B Jia Y Hong J Sun Q Gao S Hu Y. Sodium butyrate ameliorates high-fat-diet-induced non-alcoholic fatty liver disease through peroxisome proliferator-activated receptor alpha-mediated activation of beta oxidation and suppression of inflammation. J Agric Food Chem. (2018) 66:7633–42. 10.1021/acs.jafc.8b01189 29961332

[286] Mortaz E Adcock IM Folkerts G Barnes PJ Paul Vos A Garssen J. Probiotics in the management of lung diseases. Mediat Inflamm. (2013) 2013:751068. 10.1155/2013/751068 23737654 PMC3662166

[287] Matt SM Allen JM Lawson MA Mailing LJ Woods JA Johnson RW. Butyrate and dietary soluble fiber improve neuroinflammation associated with aging in mice. Front Immunol. (2018) 9:1832. 10.3389/fimmu.2018.01832 30154787 PMC6102557

[288] Lu M Wang Z. Microbiota and Aging. Adv Exp Med Biol. (2018) 1086:141–56. 10.1007/978-981-13-1117-8_9 30232757

[289] Bishehsari F Engen PA Preite NZ Tuncil YE Naqib A Shaikh M. Dietary fiber treatment corrects the composition of gut microbiota, promotes SCFA production, and suppresses colon carcinogenesis. Genes. (2018) 9:E102. 10.3390/genes9020102 29462896 PMC5852598

[290] Xu Z Tao J Chen P Chen L Sharma S Wang G. Sodium butyrate inhibits colorectal cancer cell migration by downregulating Bmi-1 through enhanced miR-200c expression. Mol Nutr Food Res. (2018) 62:e1700844. 10.1002/mnfr.201700844 29418071

